# Joint association of insulin resistance, fatty liver and fibrosis indices with MACE and mortality in individuals with Cardiovascular-Kidney-Metabolic syndrome stages 0–3

**DOI:** 10.3934/publichealth.2026041

**Published:** 2026-07-20

**Authors:** Yanqiu Huang, Wen Gu, Yadan Xu, Minxi Lin, Jun Lu, Chenghao Zhang, Yan Wu, Dengke Wang, Xia Shen, Yang Yang, Hui Wang

**Affiliations:** 1 Department of Food Hygiene and Nutrition, School of Public Health, Shanghai Jiao Tong University School of Medicine, Shanghai, China; 2 NO.2 high school of East China Normal University, Shanghai, China; 3 School of Political Science and International Relations, East China Normal University, Shanghai, China; 4 Luohe Center for Disease Control and Prevention, Luohe, Henan, China; 5 Center for Single-Cell Omics, School of Public Health, Shanghai Jiao Tong University School of Medicine, Shanghai, China; 6 Hainan International Medical Center, Shanghai Jiao Tong University School of Medicine, Hainan, China

**Keywords:** insulin resistance, fatty liver indices, fibrosis index, MACE, mortality, UK Biobank, NHANES

## Abstract

Both fatty liver/fibrosis and insulin resistance (IR) contribute to the development of Cardiovascular-Kidney-Metabolic (CKM) syndrome and elevate cardiovascular risk. This study aimed to investigate the joint associations of IR and fatty liver/fibrosis indices with major adverse cardiovascular events (MACE) and mortality in individuals with CKM syndrome stages 0–3. This study enrolled 259,699 and 6808 CKM syndrome stages 0–3 from the UK Biobank (UKB) and the National Health and Nutrition Examination Survey (NHANES). Two IR-related indices (triglyceride-glucose index, TyG; cardiometabolic index, CMI), fatty liver indices (Fatty Liver Index, FLI; Hepatic Steatosis Index, HSI), and liver fibrosis score (LiverRisk score, LRS) were calculated to construct composite indices. Multivariable Cox proportional hazards models, restricted cubic splines, Kaplan–Meier analyses, and time-dependent receiver operating characteristic curves were employed to assess prognostic associations. Mediation modeling and proteomic enrichment analyses provided a robust framework for exploring the potential pathophysiological pathways. In the UKB, elevated levels of the IR-FLI/HSI/LRS indices were significantly associated with increased risks of MACE and mortality, with consistent findings observed for all-cause and CVD mortality in the NHANES. Significant positive nonlinear relationships were established between the CMI-FLI/HSI/LRS indices and the risk of MACE. Furthermore, these composite indices demonstrated superior 10-year predictive performance compared to individual IR-related indices, and the combination of CMI and LRS showed the best performance for predicting cardiovascular mortality in both cohorts, with AUCs of 0.755 in the UKB and 0.847 in the NHANES. Mediation analysis revealed that C-reactive protein, neutrophils, and leukocytes partially mediated these associations (ratio 5.3%–14.8%). Proteomic analyses identified cytokine–cytokine receptor interactions as a primary regulatory hub, while leukocyte migration, chemotaxis, and neutrophil degranulation emerged as potential effector mechanisms implicated in this process. IR–fatty liver/fibrosis indices are significantly associated with MACE and mortality and may serve as promising biomarkers for early screening and targeted intervention in CKM stages 0–3.


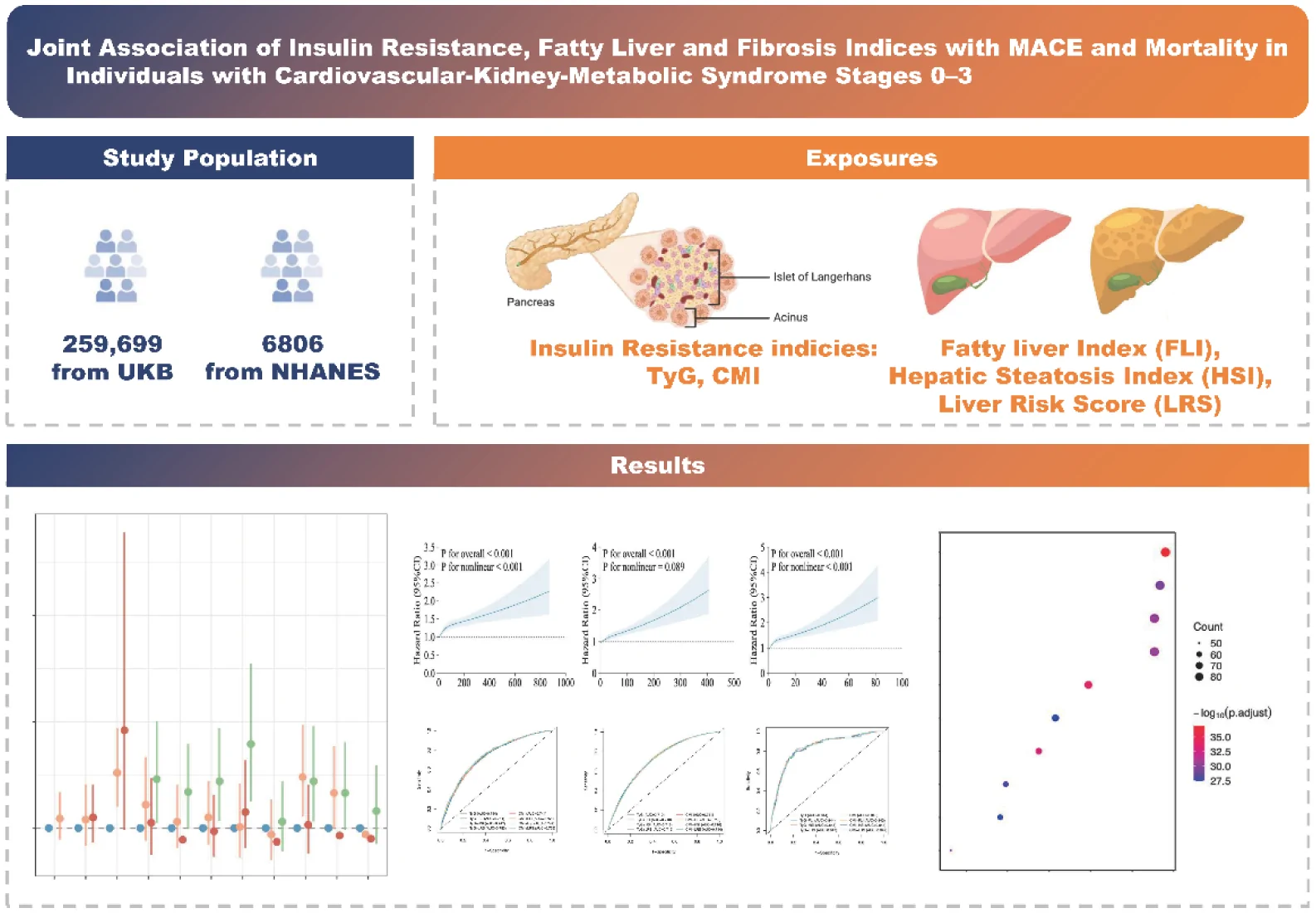


**Graphical abstract**

## 1. Introduction

Proposed by the American Heart Association (AHA) in 2023, Cardiovascular-Kidney-Metabolic (CKM) syndrome is a novel five-stage clinical entity that conceptualizes the complex interplay among metabolic disorders (e.g., obesity and diabetes), chronic kidney disease (CKD), and cardiovascular disease (CVD) [1]–[4]. The interconnected pathophysiology underlying this syndrome involves oxidative stress, chronic inflammation, and immune dysregulation, which collectively drive multi-organ damage and substantially elevate the risk of major adverse cardiovascular events (MACE) [1],[5]. However, the current CKM framework notably underestimates the pivotal role of the liver. Mounting evidence indicates that metabolic dysfunction–associated steatotic liver disease (MASLD) is not merely a frequent comorbidity but a critical catalyst accelerating CKM progression [6],[7]. As a central regulator of glucose and lipid homeostasis, the metabolically compromised liver directly fuels CKM advancement through hepatic glucose overproduction and dyslipidemia. Mechanistically intertwined with insulin resistance (IR) and obesity, this hepatic dysfunction exacerbates systemic inflammation, oxidative stress, and lipotoxicity. These factors, in turn, promote endothelial dysfunction and aggravate the pathogenesis of atherosclerosis, heart failure, and CKD [8]–[10]. Given the substantial economic burden and high mortality rates associated with CKM-related cardiovascular complications, there is an urgent clinical need to identify reliable, liver metabolism-related biomarkers [11]. Such biomarkers are essential for predicting MACE and mortality in individuals at CKM stages 0–3, thereby facilitating timely and targeted early interventions.

IR serves as a core pathological driver of CKM syndrome. Together with hepatic steatosis and fibrosis, IR reflects a profound state of systemic inflammation, adipose tissue dysfunction, and energy imbalance [12],[13]. The triglyceride-glucose (TyG) index and the cardiometabolic index (CMI) are well recognized as practical and cost-effective surrogate markers for IR, demonstrating high concordance with the gold standard hyperinsulinemic–euglycemic clamp [14],[15]. Their prognostic utility in predicting MACE and mortality among individuals with CKM syndrome stages 0–3 has been extensively validated [16],[17]. Concurrently, the detrimental impacts of excessive hepatic lipid accumulation and progressive fibrosis on vascular injury and atherogenesis have been corroborated by numerous studies [18],[19]. Specifically, elevated hepatic steatosis indices, such as the Fatty Liver Index (FLI) and Hepatic Steatosis Index (HSI), along with fibrosis metrics like LiverRisk score (LRS), are strongly associated with an increased risk of CVD [20]–[23]. Despite the complex pathophysiological crosstalk and shared inflammatory and metabolic pathways linking IR, fatty liver, and fibrosis, their synergistic impact on MACE and mortality remains unexplored. However, existing studies largely rely on individual markers that fail to fully capture the complex multi-organ crosstalk between systemic insulin resistance and hepatic metabolic dysfunction. Therefore, constructing a composite index is necessary to integrate these interconnected pathways and better reflect the combined pathological burden driving cardiovascular risk in CKM syndrome. This study aims to construct a reliable composite biomarker that integrates these interconnected factors to accurately predict MACE and mortality, thereby empowering early screening and targeted interventions.

We hypothesized that the IR-FLI/HSI and IR-LRS are significantly associated with an increased risk of MACE and mortality. Utilizing longitudinal data from two representative cohorts, namely the UK Biobank (UKB) and the National Health and Nutrition Examination Survey (NHANES), this research aimed to rigorously quantify these associations and evaluate the predictive performance of the indices for these cardiovascular outcomes.

## 2. Methods

### 2.1. Study design and study population

The study utilized data from the UKB and NHANES. In the UKB, a total of 502,248 participants were enrolled between 2006 and 2010. Participants were excluded if they had missing data required for defining CKM syndrome stages, were not classifiable into CKM syndrome stages 0 to 3, or had prevalent CVD at baseline. Individuals with missing outcome variables or covariates were also excluded, resulting in a final analytical sample of 259,699 participants (Figure S1). NHANES recruited 101,316 participants between 1998 and 2018. Following the exclusion of participants with missing data for relevant variables, 6806 participants were included in the final analysis (Figure S2). The UKB received ethical approval from the North West Multi-Center Research Ethics Committee (approved research ID: 194423, approval date: October 30, 2024). All participants gave written informed consent before enrollment in the study, which was conducted in accordance with the principles of the Declaration of Helsinki. The National Center for Health Statistics and the Ethics Review Board approved the protocol for NHANES, and all participants provided written informed consent. All participants were informed and agreed to participate in this study.

### 2.2. Definition of CKM syndrome

CKM syndrome stage 0 was defined by normal body mass index (BMI) and waist circumference (WC). Stage 1 comprised individuals with elevated BMI, increased WC, or prediabetes. Stage 2 was defined by the presence of metabolic risk factors, including elevated triglycerides, hypertension, diabetes, or metabolic syndrome, or moderate-to-high-risk chronic kidney disease. Stage 3 was characterized by very-high-risk KDIGO chronic kidney disease categories or a high predicted 10-year CVD risk. Stage 4 was identified by self-reported CVD, including coronary heart disease, angina, myocardial infarction, heart failure, and stroke [2].

### 2.3. Definition of IR-related indices, Fatty Liver Index (FLI), Hepatic Steatosis Index (HSI), and Liver Risk Score (LRS)

Two insulin resistance (IR)-related indices were calculated: the triglyceride-glucose (TyG) index and the cardiometabolic index (CMI). The TyG index was computed as Ln [fasting triglycerides (mg/dL) × fasting glucose (mg/dL) / 2]. The CMI was derived using the formula: triglycerides (mmol/L) / HDL-C (mmol/L) × waist-to-height ratio (WHTR). In both the UK Biobank and NHANES cohorts, waist circumference and height used to calculate WHTR were obtained from standardized measurements conducted at examination centers.

In the study, three fatty liver and fibrosis indices were calculated: the FLI, the HSI, and the LRS. The FLI was defined by a set of variables, including TG, BMI, gamma-glutamyl transferase (GGT), and waist circumference, with the specific formula as follows: FLI = (e^0.953 × ln(TG) + 0.139 × BMI + 0.718 × ln(GGT) + 0.053 × waist circumference − 15.745^) ÷ (1+e^0.953 × ln(TG) + 0.139 × BMI + 0.718 × ln(GGT) + 0.053 × waist circumference − 15.745^) × 100 [20]. We calculated the HSI using the following equation: HSI = 8 × (ALT/AST ratio) + BMI (+2, if diabetes; +2, if women) [22]. The definition of LRS included age, sex, fasting glucose, cholesterol, aspartate aminotransferase (AST), alanine aminotransferase (ALT), gamma-glutamyl transferase (GGT), and platelet count. Using these variables, the LiverRisk score was calculated and exported via the multiple calculations function available at https://www.liverriskscore.com/multicalc [21].

Six combined indicators were included in the analysis: TyG-FLI, TyG-HSI, TyG-LRS, CMI-FLI, CMI-HSI, and CMI-LRS. All indices were calculated by multiplying TyG or CMI by the FLI, HSI, and LRS.

### 2.4. Definition of cardiovascular events

In this study, the primary outcomes of the UKB were CVD mortality and MACE. MACE was defined as the first occurrence of any of the following during follow-up: hospitalization for acute myocardial infarction (ICD-10 codes I21 or I23), ischemic stroke (I63 or I64), intracerebral hemorrhage (I61), subarachnoid hemorrhage (I60), unstable angina (I20), or heart failure (I50); or cardiovascular death. Hospitalizations were identified using primary or secondary ICD-10 diagnosis codes from hospital episode statistics, and fatal events were ascertained from death registry data. The first nonfatal hospitalization or cardiovascular death during follow-up was considered to have met the MACE endpoint. CVD mortality was defined by the ICD-10 codes (I00–I99). In the NHANES, the follow-up period started at the time of the initial interview and concluded either at the time of death or on December 31, 2019, whichever came first, marking the cutoff for the mortality period. These files were linked to the National Death Index (NDI) using a probabilistic matching algorithm to ensure accurate mortality tracking. The causes of death were categorized according to the ICD-10, with potential underlying causes reclassified. All-cause mortality was regarded as death attributable to any cause, whereas CVD mortality included deaths attributed to heart disease (i.e., I00–I09, I11, I13, or I20–I51) or cerebrovascular disease (i.e., I60–I69) [17],[24].

### 2.5. Covariates

A set of covariates was included to account for potential confounding factors in the relationship between the indices and outcomes. Within the UKB, baseline age and sex were derived from self-reported data. Other demographic and lifestyle characteristics were gathered via touchscreen questionnaires, encompassing race (categorized as Asian, Black, White, Mixed, Chinese, or Other), education level (grouped as less than high school or high school and above), the Townsend index, smoking status (classified as never, current, or former), and daily alcohol consumption (expressed in grams per day). Medical history and medication use, including hypertension, diabetes, dyslipidemia, and the corresponding use of antihypertensive, antidiabetic, and lipid-lowering drugs, were treated as binary indicators. Two multivariable models were employed. Model 1 was adjusted for sex, age, and race. Model 2 was additionally adjusted for all other covariates listed. In the NHANES analysis, Model 1 applied identical adjustments, whereas Model 2 additionally accounted for educational level, smoking status, daily alcohol intake, income level (categorized as high, medium, or low), hypertension (yes/no), and total cholesterol.

### 2.6. Statistical analyses

Comparison of baseline demographic characteristics between study populations was conducted using independent sample t-tests for continuous variables and Pearson's chi-square tests for categorical variables. Continuous data were summarized as mean ± standard deviation (SD), whereas categorical data were presented as frequencies with corresponding percentages.

Multivariate Cox proportional hazards models were initially fitted to evaluate the associations between all the indices and cardiovascular events, with TyG-FLI, TyG-HSI, TyG-LRS, CMI-FLI, CMI-HSI, and CMI-LRS categorized into quantiles (Q1, Q2, Q3, Q4). Restricted cubic spline (RCS) curves were subsequently employed to assess the nonlinear relationships between exposure and outcomes. To improve clinical interpretability, potential cutoff points were identified from the RCS curves and marked in the revised figures. Time-dependent receiver operating characteristic (ROC) analysis was then performed to evaluate the predictive performance of the indices, where a higher area under the curve (AUC) reflected superior predictive accuracy. We also conducted stage-specific time-dependent ROC analyses in CKM stage-0 and stage-3 participants to explore whether the predictive performance of the IR-related indices and their combinations with fatty liver/fibrosis indices differed at the two extremes of the CKM spectrum. To further evaluate the incremental predictive performance of the composite indices, we calculated C-index values for individual IR-related indices and their combinations with fatty liver/fibrosis indices. The predictive performance of the composite indices was compared with that of TyG or CMI alone. In the UKB, mediation analyses were conducted to explore the potential mediating roles of C-reactive protein, neutrophils, and leukocytes in the association between the indices and cardiovascular events. To test the robustness of the results, the fatty liver indices, liver fibrosis index, and IR-related indices (TyG and CMI) were dichotomized into high and low categories and subsequently combined to form a four-group composite indicator, whose association with outcomes was analyzed. Lastly, functional enrichment analyses were carried out utilizing the Gene Ontology (GO), the Kyoto Encyclopedia of Genes and Genomes (KEGG), and the Reactome databases to identify the key signaling pathways and molecular mechanisms underlying the observed associations.

To further examine whether the associations between IR–fatty liver/fibrosis indices and cardiovascular outcomes were influenced by CKM disease severity, we performed additional stage-specific analyses among participants with CKM syndrome stages 0, 1, 2, and 3. Within each CKM stage, multivariable Cox proportional hazard models were used to evaluate the associations of tertiles of each composite index with MACE and CVD mortality, using the lowest tertile as the reference group.

Statistical analyses were performed using R (version 4.4.1) and SAS (version 9.4) for the UKB and NHANES cohorts. A two-sided P value < 0.05 was considered statistically significant for all analyses.

## 3. Results

### 3.1. Baseline characteristics of the participants

The baseline characteristics of the included individuals with CKM syndrome stages 0–3 from two national cohorts (259,699 in the UKB, 6806 in the NHANES) are summarized in Table 1. In the UKB, the mean age of participants was 56.1 years, with 48.7% being male, 92.2% White, and 82.2% having attained at least high school education. Meanwhile, the NHANES presented a higher mean age of 62.1 years, comprising 43.5% males, 58.9% White individuals, and 73.9% with a high school education or above. Compared to NHANES, the UKB participants exhibited a slightly higher smoking rate (46.4% vs. 45.5%) and daily alcohol consumption (18.6 vs. 10.7 g) but reported significantly lower hypertension prevalence (25.9% vs. 32.5%).

**Table 1.** Baseline characteristics of participants from two cohorts.

Characteristics	UK Biobank	US NHANES	P value
Participants, *N*	259,699	6806	
Sex, *N* (%)			<0.001
male	126,470 (48.7)	2959 (43.5)	
female	133,229 (51.3)	3847 (56.5)	
Age, years, mean (s.d.)	56.1 (8.0)	62.1 (10.9)	<0.001
Race, *N* (%)			<0.001
White	239,360 (92.2)	4011 (58.9)	
Other	20,339 (7.8)	2795 (41.1)	
Education level, *N* (%)			<0.001
low	46,348 (17.8)	1773 (26.1)	
high	213,351 (82.2)	5033 (73.9)	
Smoking status, *N* (%)			<0.001
No	139,270 (53.6)	3711 (54.5)	
Yes	120,429 (46.4)	3095 (45.5)	
Daily alcohol consumption, g, mean (s.d.)	18.6 (17.8)	10.7 (10.9)	<0.001
Hypertension, *N* (%)			<0.001
No	192,359 (74.1)	4593 (67.5)	
Yes	67,340 (25.9)	2213 (32.5)	
TC, mg/dl, mean (s.d.)	224.0 (42.5)	203.5 (41.0)	<0.001
TyG, mean (s.d.)	8.7 (0.6)	8.7 (0.6)	<0.001
CMI, mean (s.d.)	0.7 (0.6)	0.8 (0.8)	<0.001
FLI, mean (s.d.)	46.8 (29.7)	54.0 (30.1)	<0.001
HSI, mean (s.d.)	35.3 (5.7)	38.2 (7.2)	<0.001
LRS, mean (s.d.)	5.0 (1.2)	5.6 (1.5)	<0.001
TyG-FLI, mean (s.d.)	418.2 (278.5)	482.8 (283.0)	<0.001
TyG-HSI, mean (s.d.)	308.0 (60.7)	335.2 (74.5)	<0.001
TyG-LRS, mean (s.d.)	43.4 (11.9)	49.6 (15.4)	<0.001
CMI-FLI, mean (s.d.)	46.9 (63.1)	54.1 (75.5)	<0.001
CMI-HSI, mean (s.d.)	27.4 (27.4)	31.6 (34.6)	<0.001
CMI-LRS, mean (s.d.)	3.8 (4.1)	4.6 (5.7)	<0.001
CKM syndrome status, *N* (%)			<0.001
0	42,979 (16.5)	251 (3.7)	
1	41,905 (16.1)	864 (12.7)	
2	129,417 (49.8)	4723 (69.4)	
3	45,398 (17.5)	968 (14.2)	

Note: P values less than 0.05 (p < 0.05) were considered significant. NHANES = National Health and Nutrition Examination Survey; CKM = Cardiovascular-Kidney-Metabolic; *N* = number; s.d. = standard deviation; TyG = triglyceride-glucose; CMI = Cardiometabolic Index; FLI = Fatty Liver Index; HSI = Hepatic Steatosis Index; LRS = LiverRisk Score; HR = hazard ratio; CI = confidence interval; TC = total cholesterol.

Furthermore, NHANES participants demonstrated significantly higher levels across IR-related indices, fatty liver/fibrosis indices, and their combined indices, except TyG and TC. Regarding CKM syndrome staging, the distribution across stages 0–3 in the UKB was 16.5%, 16.1%, 49.8%, and 17.5%, respectively, whereas the corresponding proportions in NHANES were 3.7%, 12.7%, 69.4%, and 14.2%. Notably, stage 2 was the predominant category in both cohorts, accounting for approximately half or more of the respective populations.

### 3.2. Association of insulin resistance–fatty liver/fibrosis indices with MACE and mortality

Figure 1 and Tables S1–S4 present the individual and joint effects of IR and fatty liver/fibrosis indices on MACE and mortality among participants at CKM stages 0–3 in both the UKB and NHANES. Notably, elevated levels of either individual IR or fatty liver/fibrosis indices were strongly associated with an increased risk of MACE and mortality across both cohorts.

When evaluating the impact of the composite indices, specifically within the UKB cohort, higher IR-FLI levels (compared to the lowest quartile, Q1) were significantly associated with a 14%–69% increased risk of CVD mortality (HR range of Q2–Q4: TyG–FLI: 1.18–1.69; CMI–FLI: 1.14–1.50) and a 17%–60% increased risk of MACE (HR range of Q2–Q4: TyG–FLI: 1.20–1.60; CMI–FLI: 1.17–1.53; Figure 1 and Tables S1–S2). Similarly, elevated IR-HSI corresponded to a 9%–31% higher risk of CVD mortality (Q4: TyG–HSI: HR, 95% CI: 1.31, 1.21–1.42; Q3–Q4: CMI–HSI: 1.09, 1.00–1.19; 1.30, 1.20–1.42) and a 5%–40% higher risk of MACE (HR range of Q2–Q4: TyG-HSI: 1.05–1.39; CMI–HSI: 1.08–1.40). Consistent with the UKB findings, higher CMI–FLI in the NHANES significantly elevated CVD mortality risk by 44%–48%, while participants in the highest quartile (Q4) of TyG–HSI experienced a 44% increase in CVD mortality risk (1.44, 1.07–1.94; Figure 1 and Tables S3–S4).

Furthermore, in the UKB, an elevated IR-LRS was significantly linked to a 12%–42% increased risk of CVD mortality (Q3–Q4: TyG–LRS: 1.12, 1.01–1.24; 1.34, 1.21–1.48; CMI–LRS: 1.17, 1.06–1.28; 1.42, 1.29–1.56). In contrast, within NHANES, a significant 79% increase in CVD mortality risk was observed only among those in the highest TyG–LRS quartile (Q4: 1.79, 1.25–2.55). Meanwhile, compared to Q1, higher TyG–LRS and CMI–LRS conferred a 7%–22% (Q2–Q4: 1.07, 1.02–1.12; 1.13, 1.08–1.19; 1.22, 1.16–1.28) and 17%–46% (Q2–Q4: 1.17, 1.11–1.22; 1.28, 1.23–1.34; 1.46, 1.39–1.52) increased risk of MACE, respectively. Finally, in the NHANES, individuals in Q4 for TyG–LRS and CMI–LRS exhibited a 44% and 26% higher risk of all-cause mortality, respectively (TyG–LRS: 1.44, 1.19–1.74; CMI–LRS: 1.26, 1.06–1.49).

**Figure 1.**
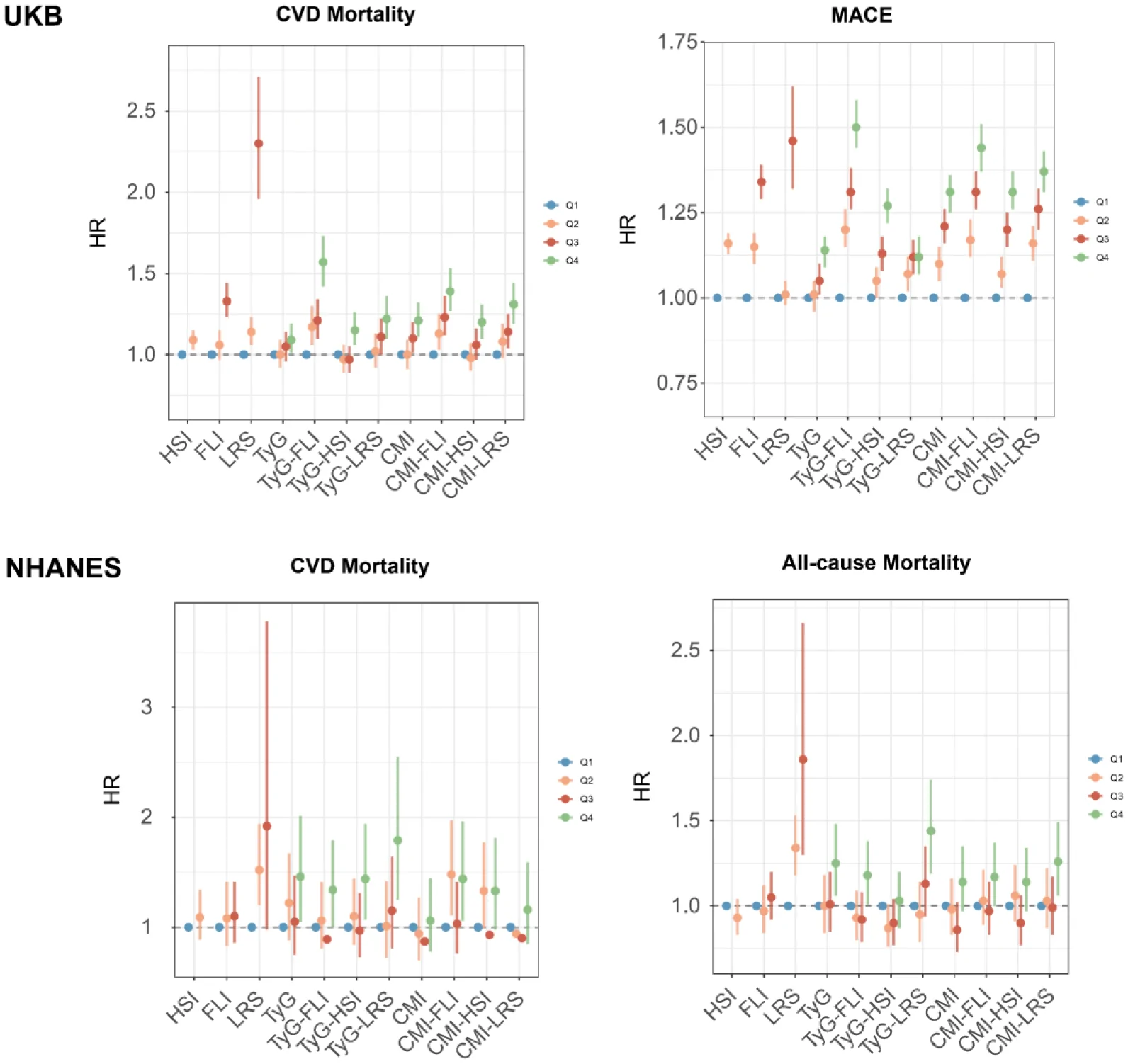
Associations of insulin resistance–fatty liver/fibrosis indices with MACE and mortality in individuals with Cardiovascular-Kidney-Metabolic syndrome stages 0–3. Note: The model was adjusted for sex, age, race, education level, income level, smoking status, daily alcohol consumption, hypertension, and TC. FLI: <30, non-FLD; 30–60, grade 1-FLD; ≥60: grade 2-FLD. HSI: <36, non-FLD; ≥36, FLD. LRS: <6, low risk; 6–10, medium risk; ≥10, high risk.

### 3.3. Association of continuous insulin resistance–fatty liver/fibrosis indices with MACE and mortality

Figure 2 and Figures S3–S5 illustrate the nonlinear relationships between the continuous indices and the risks of MACE and mortality in the UKB and NHANES. Within the UKB, with the exception of TyG–LRS, the remaining five indices demonstrated significant, positive nonlinear associations with MACE (P value < 0.05). Similarly, excluding TyG–LRS and CMI–HSI, the other four indices exhibited significant, positive nonlinear correlations with CVD mortality (P value < 0.001).

In the NHANES, consistent with the findings from the UKB, TyG–HSI maintained a significant nonlinear association with CVD mortality (P value = 0.007). Furthermore, both TyG–FLI and TyG–HSI displayed significant, positive nonlinear relationships with all-cause mortality (P value < 0.001). However, the nonlinear trends for the remaining indices did not reach statistical significance (Figure S3).

**Figure 2.**
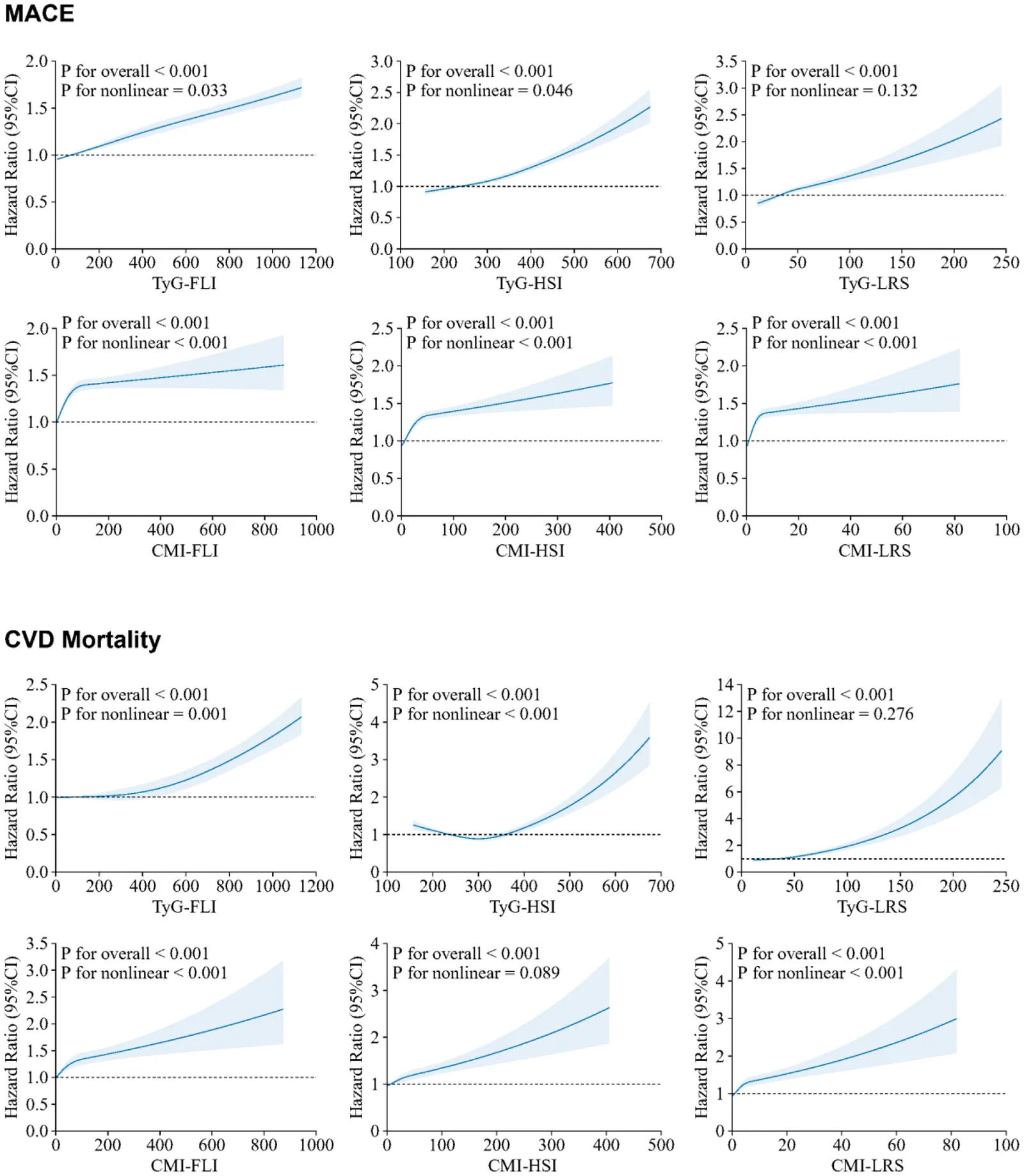
Restricted cubic spline curve illustrating the associations of insulin resistance–fatty liver/fibrosis indices with MACE and CVD mortality in the UK Biobank. Note: The model was adjusted for sex, age, race, education level, income level, smoking status, daily alcohol consumption, hypertension, and TC.

### 3.4. Cumulative incidence of MACE and mortality stratified by insulin resistance–fatty liver/fibrosis indices

Figure S6 presents the incidence rates of MACE and CVD mortality, stratified by the quartiles of the six composite indices. Within the UKB, highly significant differences were observed across the four quartile groups (P < 0.001). Specifically, a pronounced dose-response relationship was evident, wherein the incidence rates of both MACE and cardiovascular mortality increased progressively across the higher quartiles.

### 3.5. Time-dependent ROC curves of insulin resistance–fatty liver/fibrosis indices for predicting 10-year cardiovascular events

Given that IR-related indices are well-established predictors of cardiovascular events, we further evaluated the predictive performance of individual IR-related indices and their combinations with fatty liver/fibrosis indices. Across both the UKB and NHANES, we consistently observed that incorporating fatty liver/fibrosis indices led to varying degrees of improvement in the prediction of 10-year MACE and mortality (Figure 3).

Specifically, for the prediction of 10-year all-cause and CVD mortality, the combinations of TyG and CMI with the LRS demonstrated optimal predictive performance (UKB: TyG + LRS: AUC = 0.753; CMI + LRS: AUC = 0.755; NHANES: TyG/CMI + LRS: AUC = 0.856). In contrast, for MACE prediction, the combinations of TyG and CMI with the FLI achieved the best performance (AUC = 0.718).

In additional stage-specific ROC analyses, the predictive performance of the indices differed between CKM stage 0 and stage 3 participants. In CKM stage 0, TyG + LRS and CMI + LRS showed the highest AUCs for CVD mortality, both reaching 0.802, while CMI + LRS showed the highest AUC for MACE at 0.773. In CKM stage 3, CMI + LRS showed the highest AUC for CVD mortality at 0.741, whereas TyG + FLI and CMI + FLI showed the highest AUCs for MACE, both reaching 0.683. These findings suggest that the added predictive value of liver-related indices may differ across CKM stages (Table S5).

The C-index analyses showed patterns consistent with the ROC curves, indicating that the composite indices had higher predictive performance than individual IR-related indices, particularly for CMI combined with LRS in predicting CVD mortality and for FLI-based combinations in predicting MACE (Table S6).

**Figure 3.**
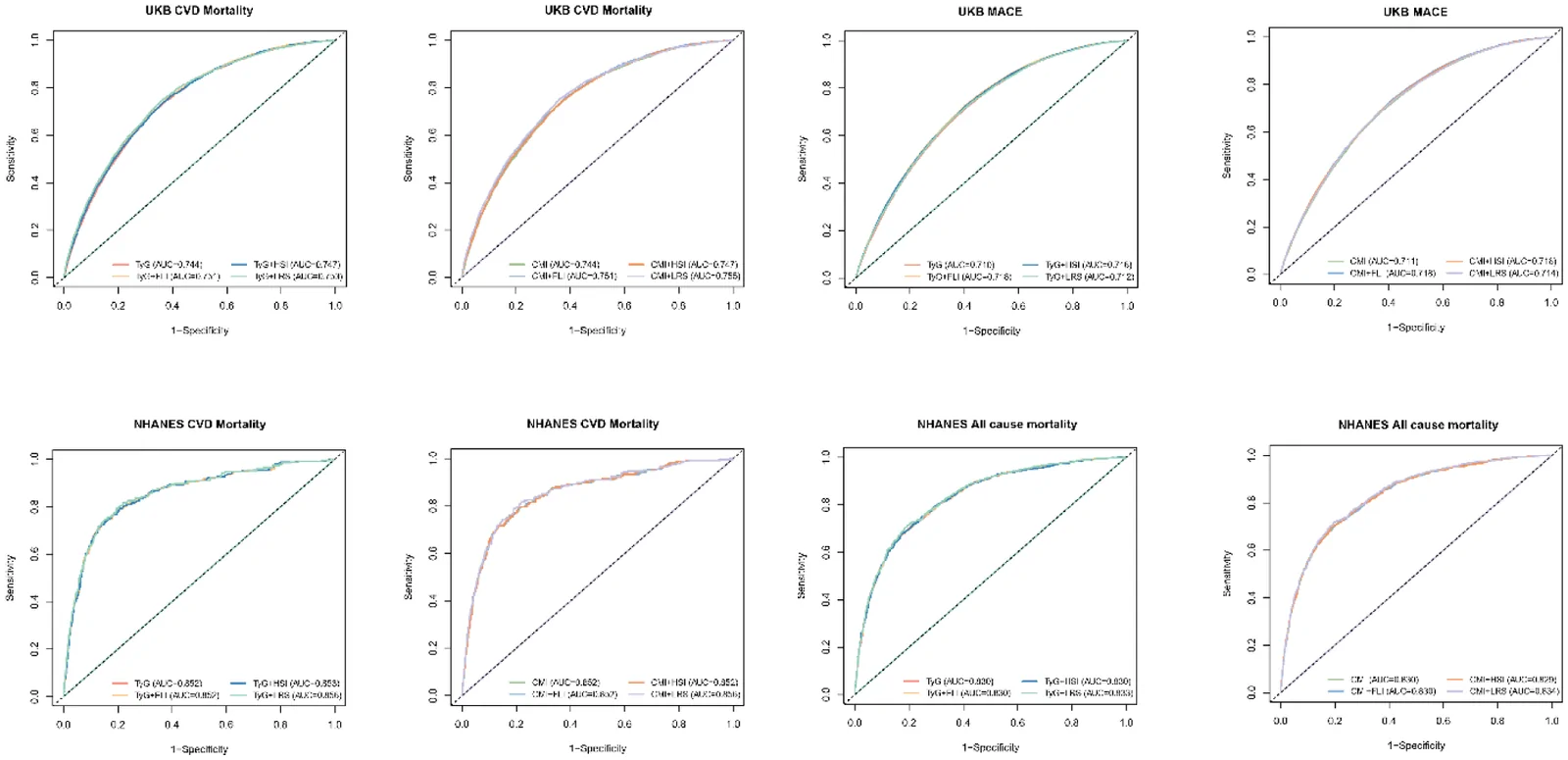
Time-dependent ROC curves of insulin resistance–fatty liver/fibrosis indices for predicting 10-year cardiovascular events. Note: The model was adjusted for sex, age, and race.

### 3.6. Mediating effects of inflammatory markers

Mediation analyses were performed to quantify the mediating role of inflammatory markers in the associations between the IR-FLI/HSI/LRS and MACE (Figure 4). CRP, neutrophils, and leucocyte mediated 5.3%–14.2% of the association between the IR–FLI/HSI and MACE (TyG–FLI: CRP: 11.4%; neutrophils: 8.2%; leucocyte: 5.3%; CMI–FLI: CRP: 13.9%; neutrophils: 12.1%; leucocyte: 8.7%; TyG–HSI: CRP: 14.2%; neutrophils: 9.5%; leucocyte: 6.1%; CMI–HSI: CRP: 13.7%; neutrophils: 12.7%; leucocyte: 9.3%). Furthermore, regarding the IR–LRS, these inflammatory mediators were found to mediate 9.0%–14.8% of the effect on MACE (TyG–LRS: CRP: 14.8%; CMI–LRS: CRP: 14.4%; neutrophils: 11.9%; leucocyte: 9.0%). These results suggest that systemic inflammation acts as a modest yet significant mediator in the pathophysiological cascade linking the IR–FLI/HSI/LRS indices to MACE.

**Figure 4.**
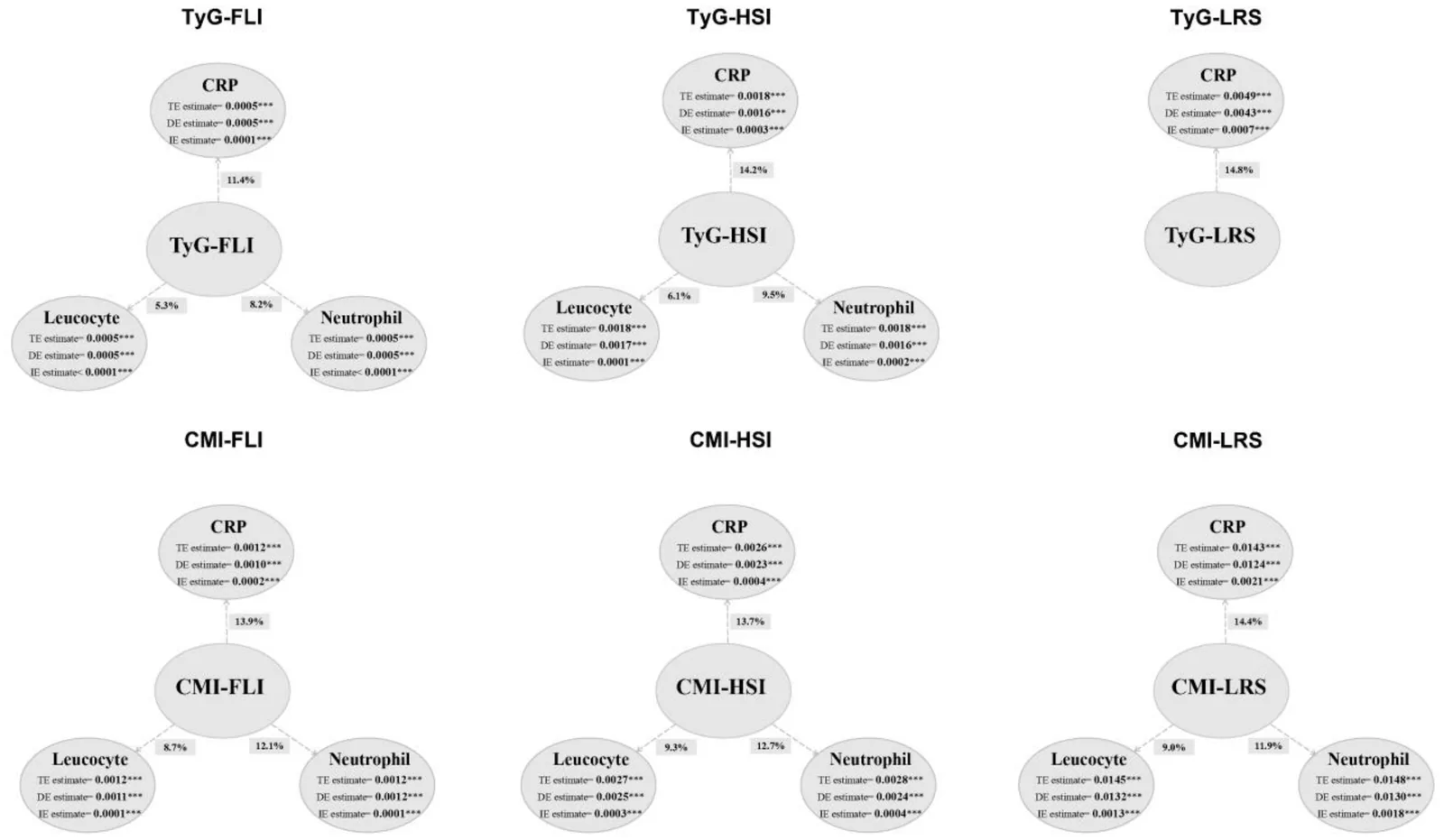
Mediation analysis of insulin resistance–fatty liver/fibrosis indices and MACE in the UK Biobank. Note: The model was adjusted for sex, age, race, education level, income level, smoking status, daily alcohol consumption, hypertension, and TC. *P < 0.05; **P < 0.01; ***P < 0.001.

### 3.7. Functional enrichment analyses of insulin resistance–fatty liver/fibrosis indices and MACE

Figure 5 illustrates the functional enrichment profiles of proteins associated with the six composite indices in relation to MACE based on GO biological processes, Reactome pathways, and KEGG analyses. In the GO analysis, significant enrichment was observed in taxis, chemotaxis, leukocyte migration, and positive regulation of cell adhesion. Similarly, Reactome pathway analysis revealed significant enrichment in neutrophil degranulation, immunoregulatory interactions between lymphoid and non-lymphoid cells, chemokine receptor signaling, and interleukin-10 signaling. Furthermore, the KEGG pathway analysis demonstrated significant enrichment in cytokine–cytokine receptor interaction, PI3K-Akt signaling, cell adhesion molecules, complement and coagulation cascades, and hematopoietic cell lineage pathways (data set). Overall, these enrichment patterns were largely shared across the six indices, indicating a convergent biological profile characterized by coordinated activation of inflammatory, immune, endothelial, and coagulation pathways.

**Figure 5.**
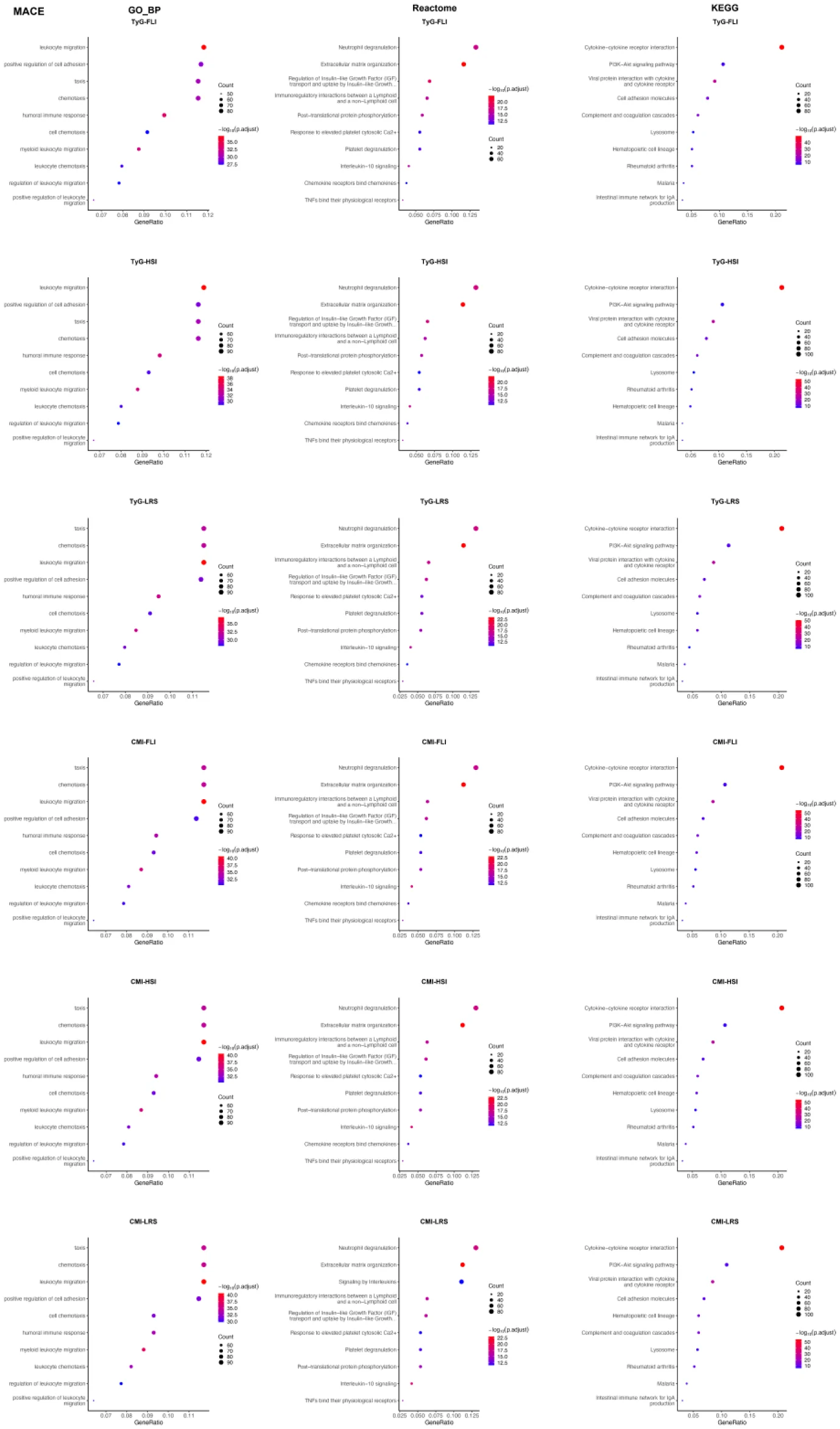
Functional enrichment analyses of insulin resistance–fatty liver/fibrosis indices and MACE utilizing the Gene Ontology, the Kyoto Encyclopedia of Genes and Genomes, and the Reactome database. Note: The model was adjusted for sex, age, race, education level, income level, smoking status, daily alcohol consumption, hypertension, and TC.

### 3.8. Sensitivity analysis

To further elucidate the synergistic effects of IR and fatty liver/fibrosis on cardiovascular events, participants were categorized into four distinct groups based on their respective index levels (Table S7). Using the subgroup with concurrently low IR and low FLI/HSI/LRS as the reference category, HRs were calculated. The results revealed that the remaining three groups exhibited significantly elevated risks for both MACE and CVD mortality to varying degrees.

We observed significant multiplicative interactions between the IR-related indices and HSI/LRS concerning the risks of MACE and CVD mortality except TyG + HSI (P value < 0.05). However, a significant multiplicative interaction between CMI and FLI was observed specifically for MACE (P value < 0.001).

In additional stage-specific analyses, the associations between composite indices and MACE were generally consistent across CKM syndrome stages 0–3 (Table S8). For TyG–FLI, the highest tertile was significantly associated with increased MACE risk in stages 0, 1, 2, and 3, with HRs of 1.33, 1.32, 1.36, and 1.22, respectively. Similar positive associations were observed for CMI–FLI, with corresponding HRs of 1.34, 1.29, 1.37, and 1.19, and for CMI–LRS, with HRs of 1.40, 1.17, 1.34, and 1.12. For CVD mortality, the associations varied across CKM stages, but remained evident, particularly in stage 2, where the highest tertiles of all six composite indices were significantly associated with increased CVD mortality risk, with HRs ranging from 1.13 to 1.40. These findings suggest that the observed associations were not solely attributable to the predominance of CKM stage-2 participants.

## 4. Discussion

To our knowledge, this study represents the first effort to evaluate the associations between the IR–FLI/HSI/LRS indices and MACE and mortality among individuals with CKM syndrome stages 0–3 across two independent cohorts. Elevated levels of these indices were significantly associated with an increased risk of MACE and mortality, with these associations remaining highly robust across all six metrics. Furthermore, significant positive nonlinear relationships were observed between these indices and the risks of MACE and CVD mortality. Mechanistically, mediation and functional enrichment analyses indicated that immune-inflammatory factors, along with their associated activation and signal transduction pathways, likely serve as the underlying molecular mechanisms linking these indices to MACE. In conclusion, integrating IR with fatty liver/fibrosis indices significantly enhances predictive performance while relying on easily accessible clinical parameters. These findings highlight the utility of these composite metrics as reliable biomarkers for early screening and targeted interventions in populations at high cardiovascular risk.

As IR-related indices, TyG and CMI reflect the systemic status of lipid and glucose metabolism. Mounting evidence has demonstrated that TyG and CMI serve as reliable biomarkers for predicting CVD and mortality in individuals with CKM syndrome stages 0–3 [15],[25]. This was corroborated by our study, where the AUC reached 0.71–0.84. Although some research suggests that combining TyG with obesity indices, such as TyG–WHTR and TyG–BMI, can effectively enhance predictive performance, these composite metrics primarily focus on phenotypic characteristics like central obesity. Consequently, they fail to reflect that lipid accumulation and IR are driven by complex multi-organ interactions. With the conceptualization of CKM syndrome, there is a growing emphasis on the intricate interplay among CVD, metabolic disorders, and CKD, which inherently involves multiple organs. A cross-sectional study of 230 patients with NAFLD in Iran indicated a positive correlation between the TyG index and liver fibrosis [26]. Another NHANES study similarly found positive associations of CMI with NAFLD and liver fibrosis [27]. The recently proposed cardiovascular-liver-kidney-metabolic (CLKM) syndrome highlights a more driving role for the liver in systemic diseases [28],[29]. The CLKM construct is a derivative definition expanded from CKM by additionally incorporating MASLD-related hepatic metabolic abnormalities, rather than a substitute for the original CKM syndrome nomenclature. As a central metabolic organ, the liver regulates lipid storage, glucose production, and detoxification [30],[31]. In MASLD, excessive fat accumulation and subsequent fibrosis impair these functions, triggering a cascade of inflammatory and lipotoxic signals. These signals can lead to endothelial dysfunction, atherosclerosis, myocardial fibrosis, and renal injury [32],[33]. Our study revealed a significant synergistic interplay between fatty liver/fibrosis and TyG/CMI, which jointly promote the occurrence of MACE and CVD mortality. Given the complex crosstalk between liver function and IR, the severity of fatty liver and fibrosis must not be overlooked when predicting cardiovascular risks.

Previous studies have established reliable composite indices based on liver function markers such as AST, ALT, and GGT to reflect fatty liver and fibrosis, including FLI, HSI, and LRS [20],[22],[23]. We constructed six composite indices combining TyG or CMI with FLI, HSI, or LRS to comprehensively capture IR, lipid metabolism, and liver function. Our findings demonstrate that higher levels of these six indices are robustly associated with an increased risk of MACE and mortality among individuals with CKM syndrome stages 0–3 in both the UKB and NHANES. Both TyG and CMI are highly accessible indicators of IR. Meanwhile, FLI allows for a more granular assessment of liver function, and HSI is well-suited for rapid community or epidemiological screening. Compared to isolated IR-related indices, these composite metrics exhibited varying degrees of enhanced predictive performance. Importantly, because both fatty liver and IR are reversible conditions, these indices hold significant clinical value for the early screening and targeted intervention of MACE risk in the population with CKM syndrome stages 0–3. The targeted interventions we envision are not limited to specific dual-target pharmacological therapies, but primarily include early lifestyle and metabolic risk management, such as weight control, dietary improvement, increased physical activity, and optimized control of glucose, lipid, blood pressure, and hepatic steatosis–related risk factors. However, whether modifying these index components can reduce subsequent cardiovascular risk requires confirmation in future prospective and interventional studies.

We acknowledge that background pharmacological treatment may influence the interpretation and predictive performance of these metabolic and hepatic biomarkers. Participants in CKM syndrome stages 2 and 3 are more likely to receive multiple medications, including lipid-lowering, antihypertensive, and antidiabetic therapies. Although we adjusted for baseline medication use in the multivariable models, these treatments may still affect several components of the composite indices. For example, statins may influence lipid profiles and liver enzyme levels, whereas antidiabetic medications may affect glucose metabolism, insulin resistance, body weight, and hepatic steatosis. Therefore, the associations between FLI, HSI, TyG, CMI, and their composite indices with cardiovascular outcomes should be interpreted in the context of background pharmacological treatment. Future studies incorporating detailed longitudinal medication information, including treatment initiation, duration, dosage, adherence, and medication changes during follow-up, are warranted to further clarify how pharmacological interventions may modify the prognostic value of these biomarkers.

CKM stage 0 and stage 3 are biologically and prognostically distinct. Stage 0 may represent an opportunity to identify early subclinical metabolic and hepatic risk, whereas stage 3 already reflects advanced CKM burden, where the incremental value of these indices may be smaller. Our additional ROC analyses showed that predictive performance was generally higher in stage 0 than in stage 3, although composite indices still modestly improved prediction in both stages. Future higher-powered studies should evaluate the prognostic value of these indices separately within each CKM stage. The distribution of CKM syndrome stages differed between the UKB and NHANES cohorts, likely reflecting differences in cohort composition, including age, sex, race, metabolic risk burden, and sampling design. To address this issue, we conducted stage-specific analyses across CKM syndrome stages 0–3. The results showed generally consistent associations with MACE across stages, suggesting that the main findings were not solely driven by the predominance of stage 2 participants. However, the associations with CVD mortality were more heterogeneous, indicating that CKM stage composition may influence the magnitude and generalizability of risk estimates.

Although we have established a strong association between the composite indices of IR and fatty liver/fibrosis with the risks of MACE and mortality, the biological processes mediating these associations remain incompletely elucidated. Our mediation analysis revealed that inflammatory markers, such as CRP, neutrophils, and leukocytes, partially mediated the cardiovascular risk conferred by these indices, with mediated proportions ranging from 5.3% to 14.8%. Notably, CRP, a key protein reflecting systemic inflammation, accounted for the highest mediation proportion, providing crucial insights into potential inflammatory pathways. Given that more than 85% of the association remained unexplained by CRP, neutrophils, and leukocytes, other biological pathways are likely to be involved. These may include insulin signaling impairment, hepatic lipotoxicity, oxidative stress, endothelial dysfunction, coagulation activation, renal metabolic disturbance, adipose tissue dysfunction, and broader liver-heart-kidney metabolic crosstalk. Therefore, the mediation findings should be interpreted as supportive evidence for a partial inflammatory pathway rather than as proof of a dominant causal mechanism.

Building upon the findings from the mediation analysis, we further conducted a proteomic enrichment analysis. Across all six indices, a highly consistent enrichment pattern emerged, predominantly involving pathways related to immune activation, inflammatory signaling, and leukocyte trafficking. In the GO biological processes, significant enrichment was observed in leukocyte migration, leukocyte chemotaxis, myeloid leukocyte migration, and the positive regulation of cell adhesion. This indicates enhanced immune cell recruitment and vascular inflammatory responses in individuals with elevated index levels. Reactome pathway analysis further revealed enrichment in neutrophil degranulation, immunoregulatory interactions between lymphoid and non-lymphoid cells, chemokine receptor signaling, and interleukin-10 signaling. These findings collectively suggest that dysregulated innate and adaptive immune interactions may be involved in the association between insulin resistance, hepatic dysfunction indices, and cardiovascular events. Consistent with these immune-related findings, KEGG pathway analysis demonstrated significant enrichment in cytokine–cytokine receptor interaction, PI3K-Akt signaling, cell adhesion molecules, complement and coagulation cascades, and hematopoietic cell lineage pathways. The involvement of complement activation and platelet degranulation pathways further implicates pro-thrombotic and endothelial dysfunction processes in the pathogenesis of MACE among individuals with higher composite index levels [34],[35]. Notably, extracellular matrix organization and post-translational protein phosphorylation pathways were also enriched, indicating potential vascular remodeling and intracellular signaling alterations. Additionally, the enrichment of insulin-like growth factor regulation and lysosome-related pathways suggests that metabolic and inflammatory crosstalk contributes to cardiovascular vulnerability. Importantly, although the magnitude of enrichment varied, these enrichment patterns were largely shared across the six indices, supporting a convergent biological framework. Overall, these findings suggest that inflammatory, immune, endothelial, and coagulation pathways may be implicated in the relationship between IR–fatty liver/fibrosis indices and MACE, although causal roles cannot be established from the present observational analysis. Importantly, these proteomic and mediation findings should be interpreted as hypothesis-generating rather than causal. Future in vivo and in vitro experimental studies, including pharmacological models targeting pathways such as PI3K-Akt signaling, cytokine–cytokine receptor interactions, leukocyte migration, and neutrophil degranulation, are needed to validate these pathophysiological cascades and determine whether they can be translated into therapeutic targets.

The present study has several notable strengths. First, this is the first investigation to utilize two independent longitudinal cohorts to propose a composite insulin resistance–fatty liver/fibrosis index as a novel biomarker for early cardiovascular screening. By integrating two sensitive surrogates of IR with two fatty liver indices and a fibrosis score, we successfully captured the synergistic interplay that drives disease trajectories. This multidimensional approach opens new avenues for comprehensive diagnostic and therapeutic strategies. Our research framework enables a highly granular assessment of both hepatic lipid accumulation and systemic metabolic dysfunction. Crucially, this dual methodological advantage relies on highly accessible metrics, making it exceptionally well-suited for large-scale epidemiological screening in community settings. Furthermore, the inclusion of diverse populations from the UKB and NHANES provides robust, cross-ethnic evidence, thereby ensuring the broad generalizability of our findings. Finally, the incorporation of proteomic and mediation analyses offers valuable mechanistic insights into potential underlying pathways, laying a solid theoretical foundation for future experimental studies.

Several limitations of the present study should be acknowledged. First, the lack of repeated measurements for the IR-FLI/HSI/LRS precluded the assessment of cumulative exposure and temporal fluctuations over the follow-up period. Second, the exclusion of participants with missing data via complete-case analysis may have introduced selection bias. Third, although we extensively adjusted for a wide range of potential covariates, the possibility of residual confounding from unmeasured factors, such as dietary patterns and physical activity, cannot be entirely ruled out. Furthermore, owing to the relatively small sample sizes in CKM stages 0 and 1, we analyzed individuals across stages 0–3 as a single aggregated cohort. This approach might obscure stage-specific nuances and limit the formulation of highly precise, targeted interventions. In addition, although CRP, neutrophils, and leukocytes showed statistically significant mediation effects, the mediated proportions were modest. This indicates that most of the association remained unexplained, and unmeasured mediators or residual confounding may have contributed to the observed relationships. Finally, the observational nature of the study design inherently precludes the establishment of definitive causal relationships. Future investigations incorporating Mendelian randomization analyses and experimental mechanistic studies are warranted to validate these findings and elucidate the underlying biological pathways.

## 5. Conclusions

Across these two longitudinal cohorts, elevated combined IR and fatty liver/fibrosis indices were robustly associated with heightened risks of MACE and mortality among patients in CKM syndrome stages 0–3, demonstrating incremental predictive value. While systemic inflammation and immune-related signaling pathways offer a plausible mechanistic framework, future studies should emphasize the serial evaluation of this composite metric and further elucidate its functional mechanisms. Our results position the IR-liver related index as a potent tool for risk stratification and a clinical biomarker for early targeted intervention in the CKM syndrome stages 0–3.

## Use of AI tools declaration

The authors declare they have not used Artificial Intelligence (AI) tools in the creation of this article.


