# Supplementary material for: Joint association of insulin resistance, fatty liver and fibrosis indices with MACE and mortality in individuals with Cardiovascular-Kidney-Metabolic syndrome stages 0–3
Source: AIMS Public Health. 2026 Jul 20;13(3):761–82. doi: 10.3934/publichealth.2026041 (PMC13684069; doi:10.3934/publichealth.2026041)
Supplement: Supplementary file 1 [file publichealth-13-03-041-s001.pdf]

Public Health

AIMS Public Health, 13(3): 761–782.
DOI: 10.3934/publichealth.2026041
Received: 27 April 2026
Revised: 18 June 2026
Accepted: 02 July 2026
Published: 20 July 2026

http://www.aimspress.com/journal/aimsph

Research article

Joint association of insulin resistance, fatty liver and fibrosis indices with MACE and mortality in individuals with Cardiovascular-Kidney-Metabolic syndrome stages 0–3

Yanqiu Huang[1,†], Wen Gu[1,†], Yadan Xu[1,†], Minxi Lin[2,†], Jun Lu[3,†], Chenghao Zhang[1], Yan Wu[1], Dengke Wang[4,*], Xia Shen[5,*], Yang Yang[1,*] and Hui Wang[1,6,*]

1 Department of Food Hygiene and Nutrition, School of Public Health, Shanghai Jiao Tong University School of Medicine, Shanghai, China
2 NO.2 high school of East China Normal University, Shanghai, China
3 School of Political Science and International Relations, East China Normal University, Shanghai, China
4 Luohe Center for Disease Control and Prevention, Luohe, Henan, China
5 Center for Single-Cell Omics, School of Public Health, Shanghai Jiao Tong University School of Medicine, Shanghai, China
6 Hainan International Medical Center, Shanghai Jiao Tong University School of Medicine, Hainan, China

* **Correspondence:** Hui Wang: Email: huiwang@shsmu.edu.cn; Yang Yang: Email: yyang93@shsmu.edu.cn; Xia Shen: Email: shenxia@shsmu.edu.cn; Dengke Wang: Email: lhzf2011@sina.com.

† These authors contributed equally.

Supplementary

Table S1. Associations of insulin resistance-fatty liver/fibrosis indices with CVD mortality in individuals with Cardiovascular-Kidney-Metabolic syndrome stages 0–3 in the UK Biobank.

Variable	Range	N (events)	Model 1		Model 2	
			HR (95% CI)	P value	HR (95% CI)	P value
**TyG**						
Q1	6.3–8.3	64,925 (901)	1	Ref	1	Ref
Q2	8.3–8.7	64,925 (1206)	1.04 (0.95,1.13)	0.412	1.01 (0.93,1.10)	0.806
Q3	8.7–9.1	64,924 (1510)	1.16 (1.07,1.26)	<0.001	1.07 (0.99,1.16)	0.106
Q4	9.1–12.5	64,925 (1929)	1.43 (1.32,1.55)	<0.001	1.18 (1.09,1.28)	<0.001
**TyG-FLI**						
Q1	1.4–160.7	64,925 (614)	1	Ref	1	Ref
Q2	160.7–384.9	64,925 (1130)	1.24 (1.13,1.37)	<0.001	1.18 (1.06,1.30)	0.002
Q3	384.9–658.8	64,924 (1483)	1.42 (1.29,1.57)	<0.001	1.23 (1.11,1.36)	<0.001
Q4	658.8–1180.6	64,925 (2319)	2.27 (2.07,2.50)	<0.001	1.69 (1.54,1.87)	<0.001
**TyG-HSI**						
Q1	147–263.6	64,925 (972)	1	Ref	1	Ref
Q2	263.6–299.2	64,925 (1206)	1.03 (0.95,1.12)	0.505	0.98 (0.90,1.06)	0.613
Q3	299.2–343.4	64,924 (1412)	1.14 (1.05,1.23)	0.002	1.00 (0.92,1.08)	0.920
Q4	343.4–826	64,925 (1956)	1.73 (1.60,1.87)	<0.001	1.31 (1.21,1.42)	<0.001
**TyG-LRS**						
Q1	0.9–36.4	64,925 (630)	1	Ref	1	Ref
Q2	36.4–41.4	64,925 (937)	1.05 (0.94,1.16)	0.383	1.02 (0.92,1.13)	0.735
Q3	41.4–47.6	64,924 (1449)	1.21 (1.09,1.34)	<0.001	1.12 (1.01,1.24)	0.030
Q4	47.6–290.5	64,925 (2530)	1.77 (1.60,1.95)	<0.001	1.34 (1.21,1.48)	<0.001
**CMI**						
Q1	0–0.3	64,925 (830)	1	Ref	1	Ref
Q2	0.3–0.5	64,925 (1140)	1.04 (0.95,1.14)	0.356	1.01 (0.92,1.10)	0.914
Q3	0.5–0.9	64,924 (1547)	1.25 (1.15,1.36)	<0.001	1.13 (1.04,1.23)	0.005
Q4	0.9–14.7	64,925 (2029)	1.57 (1.44,1.71)	<0.001	1.30 (1.19,1.41)	<0.001
**CMI-FLI**						
Q1	0–6.3	64,925 (653)	1	Ref	1	Ref
Q2	6.3–23.6	64,925 (1158)	1.21 (1.10,1.34)	<0.001	1.14 (1.03,1.25)	0.011
Q3	23.6–63.2	64,924 (1596)	1.47 (1.34,1.62)	<0.001	1.26 (1.15,1.39)	<0.001
Q4	63.2–1343.3	64,925 (2139)	1.96 (1.79,2.15)	<0.001	1.50 (1.37,1.65)	<0.001
**CMI-HSI**						
Q1	0.5–10.2	64,925 (846)	1	Ref	1	Ref
Q2	10.2–18.7	64,925 (1139)	1.03 (0.94,1.13)	0.500	0.99 (0.90,1.08)	0.750
Q3	18.7–34.6	64,924 (1517)	1.21 (1.11,1.32)	<0.001	1.09 (1.00,1.19)	0.049
Q4	34.6–569.7	64,925 (2044)	1.63 (1.50,1.77)	<0.001	1.30 (1.20,1.42)	<0.001

Continued on next page

Variable	Range	N (events)	Model 1		Model 2	
			HR (95% CI)	P value	HR (95% CI)	P value
**CMI-LRS**						
Q1	0–1.4	64,925 (698)	1	Ref	1	Ref
Q2	1.4–2.6	64,925 (1110)	1.15 (1.04,1.26)	0.005	1.09 (0.99,1.19)	0.095
Q3	2.6–4.7	64,924 (1494)	1.32 (1.20,1.44)	<0.001	1.17 (1.06,1.28)	0.001
Q4	4.7–148.8	64,925 (2244)	1.83 (1.67,2.00)	<0.001	1.42 (1.29,1.56)	<0.001
**FLI**						
Non-FLD	0.2–30.0	95,776 (1126)	1	Ref	1	Ref
Grade 1-FLD	30.0–60.0	68,870 (1361)	1.16 (1.07,1.25)	<0.001	1.06 (0.98,1.15)	0.132
Grade 2-FLD	60.0–100.0	95,053 (3059)	1.82 (1.69,1.95)	<0.001	1.42 (1.31,1.53)	<0.001
**HSI**						
Non-FLD	17.8–36.0	158,815 (2907)	1	Ref	1	Ref
FLD	36.0–86.2	100,884 (2639)	1.44 (1.37,1.52)	<0.001	1.20 (1.13,1.27)	<0.001
**LRS**						
Low risk	0.1–6.0	229,593 (4094)	1	Ref	1	Ref
Medium risk	6.0–10.0	28,273 (1274)	1.62 (1.52,1.73)	<0.001	1.26 (1.18,1.36)	<0.001
High risk	10.0–28.9	1833 (178)	4.78 (4.11,5.56)	<0.001	2.80 (2.38,3.28)	<0.001

Note: Model 1: adjusted for sex, age, race. Model 2: adjusted for sex, age, race, education level, Townsend index, smoking status, daily alcohol consumption, hypertension, dyslipidemia, antihypertensive, antidiabetic, lipid-lowering medications. FLI: < 30: Non-FLD, 30–60: Grade 1-FLD, ≥ 60: Grade 2-FLD; HSI: < 36: Non-FLD, ≥ 36: FLD; LRS: < 6: Low risk, 6–10: Medium risk, ≥ 10: High risk. P-values less than 0.05 ($p < 0.05$) were considered significant. Abbreviations: CVD = Cardiovascular Disease, FLD = Fatty Liver Disease, Ref = reference, Q = quartile, N = number, TyG = triglyceride-glucose, CMI = Cardiometabolic Index, FLI = Fatty Liver Index, HIS = Hepatic Steatosis Index, LRS = LiverRisk Score, HR = hazard ratio, CI = confidence interval.

Table S2. Associations of insulin resistance-fatty liver/fibrosis indices with MACE in individuals with Cardiovascular-Kidney-Metabolic syndrome stages 0–3 in the UK Biobank.

Variable	Range	N (events)	Model 1		Model 2	
			HR (95% CI)	P value	HR (95% CI)	P value
**TyG**						
Q1	6.3–8.3	64,925 (3950)	1	Ref	1	Ref
Q2	8.3–8.7	64,925 (5149)	1.07 (1.02,1.11)	0.002	1.01 (0.97,1.05)	0.607
Q3	8.7–9.1	64,924 (6462)	1.23 (1.18,1.28)	<0.001	1.07 (1.03,1.12)	0.001
Q4	9.1–12.5	64,925 (8294)	1.54 (1.48,1.60)	<0.001	1.21 (1.16,1.25)	<0.001
**TyG-FLI**						
Q1	1.4–160.7	64,925 (2881)	1	Ref	1	Ref
Q2	160.7–384.9	64,925 (5045)	1.31 (1.25,1.38)	<0.001	1.20 (1.15,1.26)	<0.001
Q3	384.9–658.8	64,924 (6768)	1.60 (1.53,1.67)	<0.001	1.33 (1.27,1.39)	<0.001
Q4	658.8–1180.6	64,925 (9161)	2.22 (2.12,2.32)	<0.001	1.60 (1.53,1.68)	<0.001
**TyG-HSI**						
Q1	147–263.6	64,925 (3952)	1	Ref	1	Ref
Q2	263.6–299.2	64,925 (5216)	1.14 (1.10,1.19)	<0.001	1.05 (1.01,1.09)	0.027
Q3	299.2–343.4	64,924 (6430)	1.35 (1.30,1.40)	<0.001	1.15 (1.10,1.20)	<0.001
Q4	343.4–826	64,925 (8257)	1.87 (1.80,1.94)	<0.001	1.39 (1.33,1.44)	<0.001
**TyG-LRS**						
Q1	0.9–36.4	64,925 (3328)	1	Ref	1	Ref
Q2	36.4–41.4	64,925 (4683)	1.06 (1.02,1.11)	0.007	1.07 (1.02,1.12)	0.003
Q3	41.4–47.6	64,924 (6564)	1.18 (1.13,1.23)	<0.001	1.13 (1.08,1.19)	<0.001
Q4	47.6–290.5	64,925 (9280)	1.47 (1.40,1.53)	<0.001	1.22 (1.16,1.28)	<0.001
**CMI**						
Q1	0–0.3	64,925 (3471)	1	Ref	1	Ref
Q2	0.3–0.5	64,925 (5045)	1.19 (1.14,1.24)	<0.001	1.10 (1.06,1.15)	<0.001
Q3	0.5–0.9	64,924 (6725)	1.45 (1.39,1.51)	<0.001	1.23 (1.18,1.29)	<0.001
Q4	0.9–14.7	64,925 (8614)	1.81 (1.73,1.88)	<0.001	1.38 (1.32,1.44)	<0.001
**CMI-FLI**						
Q1	0–6.3	64,925 (2968)	1	Ref	1	Ref
Q2	6.3–23.6	64,925 (5046)	1.29 (1.23,1.35)	<0.001	1.17 (1.12,1.23)	<0.001
Q3	23.6–63.2	64,924 (6903)	1.62 (1.55,1.69)	<0.001	1.34 (1.28,1.40)	<0.001
Q4	63.2–1343.3	64,925 (8938)	2.11 (2.01,2.20)	<0.001	1.53 (1.46,1.60)	<0.001
**CMI-HSI**						
Q1	0.5–10.2	64,925 (3491)	1	Ref	1	Ref
Q2	10.2–18.7	64,925 (4996)	1.17 (1.12,1.23)	<0.001	1.08 (1.03,1.13)	0.001
Q3	18.7–34.6	64,924 (6693)	1.44 (1.38,1.50)	<0.001	1.22 (1.17,1.28)	<0.001
Q4	34.6–569.7	64,925 (8675)	1.87 (1.80,1.95)	<0.001	1.40 (1.34,1.46)	<0.001

Continued on next page

Variable	Range	N (events)	Model 1		Model 2	
			HR (95% CI)	P value	HR (95% CI)	P value
**CMI-LRS**						
Q1	0–1.4	64,925 (3108)	1	Ref	1	Ref
Q2	1.4–2.6	64,925 (4978)	1.27 (1.21,1.33)	<0.001	1.17 (1.11,1.22)	<0.001
Q3	2.6–4.7	64,924 (6682)	1.52 (1.45,1.59)	<0.001	1.28 (1.23,1.34)	<0.001
Q4	4.7–148.8	64,925 (9087)	1.97 (1.88,2.06)	<0.001	1.46 (1.39,1.52)	<0.001
**FLI**						
Non-FLD	0.2–30.0	95,776 (5114)	1	Ref	1	Ref
Grade 1-FLD	30.0–60.0	68,870 (6230)	1.29 (1.24,1.34)	<0.001	1.15 (1.11,1.20)	<0.001
Grade 2-FLD	60.0–100.0	95,053 (12,511)	1.84 (1.78,1.91)	<0.001	1.41 (1.36,1.46)	<0.001
**HSI**						
Non-FLD	17.8–36.0	158,815 (12,376)	1	Ref	1	Ref
FLD	36.0–86.2	100,884 (11,479)	1.48 (1.44,1.52)	<0.001	1.24 (1.21,1.28)	<0.001
**LRS**						
Low risk	0.1–6.0	229,593 (19,050)	1	Ref	1	Ref
Medium risk	6.0–10.0	28,273 (4389)	1.32 (1.27,1.36)	<0.001	1.11 (1.07,1.15)	<0.001
High risk	10.0–28.9	1833 (416)	2.54 (2.30,2.80)	<0.001	1.76 (1.59,1.94)	<0.001

Note: Model 1: adjusted for sex, age, race. Model 2: adjusted for sex, age, race, education level, Townsend index, smoking status, daily alcohol consumption, hypertension, dyslipidemia, antihypertensive, antidiabetic, lipid-lowering medications. FLI: < 30: Non-FLD, 30–60: Grade 1-FLD, ≥ 60: Grade 2-FLD; HSI: < 36: Non-FLD, ≥ 36: FLD; LRS: < 6: Low risk, 6–10: Medium risk, ≥ 10: High risk. P-values less than 0.05 ($p < 0.05$) were considered significant. Abbreviations: MACE = major adverse cardiovascular events, FLD = Fatty Liver Disease, Ref = reference, Q = quartile, N = number, TyG = triglyceride-glucose, CMI = Cardiometabolic Index, FLI = Fatty Liver Index, HIS = Hepatic Steatosis Index, LRS = Liver Risk Score, HR = hazard ratio, CI = confidence interval.

Table S3. Associations of insulin resistance-fatty liver/fibrosis indices with CVD mortality in individuals with Cardiovascular-Kidney-Metabolic syndrome stages 0–3 in the NHANES.

Variable	Range	N (events)	Model 1		Model 2	
			HR (95% CI)	P value	HR (95% CI)	P value
**TyG**						
Q1	5.6–8.3	1702 (61)	1	Ref	1	Ref
Q2	8.3–8.7	1701 (110)	1.27 (0.93,1.74)	0.138	1.22 (0.88,1.67)	0.231
Q3	8.7–9.1	1701 (95)	1.12 (0.81,1.55)	0.492	1.05 (0.75,1.47)	0.765
Q4	9.1–12	1702 (131)	1.64 (1.20,2.24)	0.002	1.46 (1.05,2.01)	0.023
**TyG-FLI**						
Q1	5.9–229	1702 (94)	1	Ref	1	Ref
Q2	229.2–481.9	1701 (109)	1.07 (0.81,1.42)	0.612	1.06 (0.81,1.41)	0.660
Q3	482–735	1701 (89)	0.94 (0.70,1.26)	0.697	0.89 (0.66,1.19)	0.425
Q4	735–1187.7	1702 (105)	1.51 (1.13,2.01)	0.005	1.34 (1.00,1.79)	0.051
**TyG-HSI**						
Q1	159.9–281.6	1702 (106)	1	Ref	1	Ref
Q2	281.6–325.1	1701 (109)	1.10 (0.84,1.45)	0.469	1.10 (0.84,1.44)	0.482
Q3	325.1–379.8	1701 (85)	1.04 (0.78,1.39)	0.806	0.97 (0.73,1.31)	0.862
Q4	379.8–710.6	1702 (97)	1.61 (1.20,2.15)	0.001	1.44 (1.07,1.94)	0.016
**TyG-LRS**						
Q1	20.7–40.7	1702 (58)	1	Ref	1	Ref
Q2	40.7–46.5	1701 (88)	0.97 (0.69,1.37)	0.883	1.01 (0.72,1.42)	0.960
Q3	46.5–53.7	1701 (109)	1.09 (0.78,1.53)	0.618	1.15 (0.81,1.64)	0.427
Q4	53.7–332.7	1702 (142)	1.74 (1.25,2.42)	0.001	1.79 (1.25,2.55)	0.001
**CMI**						
Q1	0–0.3	1702 (75)	1	Ref	1	Ref
Q2	0.3–0.6	1701 (110)	1.03 (0.77,1.38)	0.845	0.94 (0.70,1.27)	0.690
Q3	0.6–1	1701 (100)	0.98 (0.73,1.33)	0.904	0.87 (0.64,1.18)	0.360
Q4	1–9.9	1702 (112)	1.28 (0.95,1.73)	0.109	1.06 (0.78,1.44)	0.713
**CMI-FLI**						
Q1	0–10	1702 (75)	1	Ref	1	Ref
Q2	10–29.9	1701 (123)	1.51 (1.13,2.02)	0.005	1.48 (1.11,1.97)	0.008
Q3	29.9–69.9	1701 (89)	1.10 (0.81,1.50)	0.533	1.03 (0.76,1.41)	0.838
Q4	69.9–986.5	1702 (110)	1.66 (1.22,2.24)	0.001	1.44 (1.06,1.96)	0.020
**CMI-HSI**						
Q1	1.2–11.8	1702 (75)	1	Ref	1	Ref
Q2	11.8–21.2	1701 (124)	1.40 (1.05,1.86)	0.023	1.33 (0.99,1.77)	0.056
Q3	21.2–38.9	1701 (88)	1.02 (0.74,1.39)	0.917	0.93 (0.68,1.27)	0.651
Q4	38.9–421.1	1702 (110)	1.58 (1.16,2.13)	0.003	1.33 (0.98,1.81)	0.071

Continued on next page

Variable	Range	N (events)	Model 1		Model 2	
			HR (95% CI)	P value	HR (95% CI)	P value
**CMI-LRS**						
Q1	0.2–1.7	1702 (72)	1	Ref	1	Ref
Q2	1.7–3	1701 (102)	1.02 (0.75,1.38)	0.921	0.94 (0.69,1.27)	0.678
Q3	3–5.5	1701 (102)	1.00 (0.74,1.36)	0.997	0.90 (0.66,1.22)	0.489
Q4	5.5–103.4	1702 (121)	1.38 (1.02,1.87)	0.037	1.16 (0.85,1.59)	0.338
**FLI**						
Non-FLD	0.85–30.0	1897 (106)	1	Ref	1	Ref
Grade 1-FLD	30.0–60.0	1802 (118)	1.10 (0.85,1.44)	0.459	1.08 (0.83,1.41)	0.550
Grade 2-FLD	60.0–100.0	3107 (173)	1.21 (0.94,1.54)	0.138	1.10 (0.86,1.41)	0.448
**HSI**						
Non-FLD	20.0–36.0	2900 (196)	1	Ref	1	Ref
FLD	36.0–78.0	3906 (201)	1.17 (0.95,1.44)	0.137	1.09 (0.89,1.34)	0.415
**LRS**						
Low risk	2.3–6.0	4946 (248)	1	Ref	1	Ref
Medium risk	6.0–10.0	1742 (140)	1.50 (1.20,1.87)	<0.001	1.52 (1.20,1.94)	0.001
High risk	10.0–33.9	118 (9)	2.14 (1.09,4.19)	0.026	1.92 (0.98,3.78)	0.057

Note: Model 1: adjusted for sex, age, race. Model 2: adjusted for sex, age, race, education level, income level, smoking status, daily alcohol consumption, hypertension, TC. FLI: < 30: Non-FLD, 30–60: Grade 1-FLD, ≥ 60: Grade 2-FLD; HSI: < 36: Non-FLD, ≥ 36: FLD; LRS: < 6: Low risk, 6–10: Medium risk, ≥ 10: High risk. P-values less than 0.05 ($p < 0.05$) were considered significant. Abbreviations: CVD = Cardiovascular Disease, NHANES = National Health and Nutrition Examination Survey, FLD = Fatty Liver Disease, Ref = reference, Q = quartile, N = number, TyG = triglyceride-glucose, CMI = Cardiometabolic Index, FLI = Fatty Liver Index, HIS = Hepatic Steatosis Index, LRS = Liver Risk Score, HR = hazard ratio, CI = confidence interval, TC = Total cholesterol.

Table S4. Associations of insulin resistance-fatty liver/fibrosis indices with all-cause mortality in individuals with Cardiovascular-Kidney-Metabolic syndrome stages 0–3 in the NHANES.

Variable	Range	N (events)	Model 1		Model 2	
			HR (95% CI)	P value	HR (95% CI)	P value
**TyG**						
Q1	5.6–8.3	1702 (232)	1	Ref	1	Ref
Q2	8.3–8.7	1701 (337)	1.01 (0.86,1.20)	0.870	1.00 (0.84,1.18)	0.985
Q3	8.7–9.1	1701 (358)	1.05 (0.89,1.24)	0.581	1.01 (0.85,1.20)	0.920
Q4	9.1–12	1702 (447)	1.35 (1.15,1.59)	<0.001	1.25 (1.06,1.48)	0.009
**TyG-FLI**						
Q1	5.9–229	1702 (336)	1	Ref	1	Ref
Q2	229.2–481.9	1701 (344)	0.94 (0.81,1.09)	0.414	0.93 (0.80,1.09)	0.372
Q3	482–735	1701 (338)	0.97 (0.84,1.13)	0.731	0.92 (0.79,1.08)	0.314
Q4	735–1187.7	1702 (356)	1.30 (1.12,1.52)	0.001	1.18 (1.01,1.38)	0.040
**TyG-HSI**						
Q1	159.9–281.6	1702 (404)	1	Ref	1	Ref
Q2	281.6–325.1	1701 (344)	0.87 (0.75,1.01)	0.062	0.87 (0.76,1.01)	0.067
Q3	325.1–379.8	1701 (323)	0.95 (0.82,1.10)	0.513	0.90 (0.77,1.04)	0.152
Q4	379.8–710.6	1702 (303)	1.13 (0.97,1.33)	0.117	1.03 (0.87,1.20)	0.753
**TyG-LRS**						
Q1	20.7–40.7	1702 (217)	1	Ref	1	Ref
Q2	40.7–46.5	1701 (302)	0.95 (0.79,1.13)	0.541	0.95 (0.79,1.14)	0.571
Q3	46.5–53.7	1701 (396)	1.12 (0.94,1.34)	0.196	1.13 (0.94,1.35)	0.207
Q4	53.7–332.7	1702 (459)	1.55 (1.30,1.84)	<0.001	1.44 (1.19,1.74)	<0.001
**CMI**						
Q1	0–0.3	1702 (252)	1	Ref	1	Ref
Q2	0.3–0.6	1701 (366)	1.05 (0.89,1.23)	0.572	0.98 (0.83,1.16)	0.825
Q3	0.6–1	1701 (336)	0.96 (0.81,1.13)	0.610	0.86 (0.73,1.02)	0.088
Q4	1–9.9	1702 (420)	1.33 (1.13,1.56)	0.001	1.14 (0.97,1.35)	0.118
**CMI-FLI**						
Q1	0–10	1702 (299)	1	Ref	1	Ref
Q2	10–29.9	1701 (347)	1.06 (0.91,1.24)	0.429	1.03 (0.89,1.21)	0.667
Q3	29.9–69.9	1701 (344)	1.04 (0.89,1.21)	0.665	0.97 (0.83,1.14)	0.717
Q4	69.9–986.5	1702 (384)	1.32 (1.13,1.54)	0.001	1.17 (1.00,1.37)	0.057
**CMI-HSI**						
Q1	1.2–11.8	1702 (281)	1	Ref	1	Ref
Q2	11.8–21.2	1701 (375)	1.12 (0.96,1.31)	0.147	1.06 (0.91,1.24)	0.458
Q3	21.2–38.9	1701 (335)	0.99 (0.84,1.16)	0.858	0.90 (0.77,1.06)	0.223
Q4	38.9–421.1	1702 (383)	1.32 (1.13,1.54)	0.001	1.14 (0.97,1.34)	0.123

Continued on next page

Variable	Range	N (events)	Model 1		Model 2	
			HR (95% CI)	P value	HR (95% CI)	P value
**CMI-LRS**						
Q1	0.2–1.7	1702 (230)	1	Ref	1	Ref
Q2	1.7–3	1701 (347)	1.11 (0.93,1.31)	0.241	1.03 (0.87,1.22)	0.725
Q3	3–5.5	1701 (361)	1.09 (0.92,1.29)	0.296	0.99 (0.83,1.17)	0.902
Q4	5.5–103.4	1702 (436)	1.47 (1.25,1.74)	<0.001	1.26 (1.06,1.49)	0.007
**FLI**						
Non-FLD	0.85–30.0	1897 (379)	1	Ref	1	Ref
Grade 1-FLD	30.0–60.0	1802 (380)	0.99 (0.85,1.14)	0.838	0.97 (0.84,1.12)	0.670
Grade 2-FLD	60.0–100.0	3107 (615)	1.13 (0.99,1.29)	0.060	1.05 (0.92,1.20)	0.482
**HSI**						
Non-FLD	20.0–36.0	2900 (709)	1	Ref	1	Ref
FLD	36.0–78.0	3906 (665)	1.00 (0.90,1.12)	0.992	0.93 (0.83,1.04)	0.209
**LRS**						
Low risk	2.3–6.0	4946 (878)	1	Ref	1	Ref
Medium risk	6.0–10.0	1742 (464)	1.43 (1.27,1.61)	<0.001	1.34 (1.18,1.53)	<0.001
High risk	10.0–33.9	118 (32)	2.17 (1.52,3.10)	<0.001	1.86 (1.30,2.66)	0.001

Note: Model 1: adjusted for sex, age, race. Model 2: adjusted for sex, age, race, education level, income level, smoking status, daily alcohol consumption, hypertension, TC. FLI: < 30: Non-FLD, 30–60: Grade 1-FLD, ≥ 60: Grade 2-FLD; HSI: < 36: Non-FLD, ≥ 36: FLD; LRS: < 6: Low risk, 6–10: Medium risk, ≥ 10: High risk. P-values less than 0.05 ($p < 0.05$) were considered significant. Abbreviations: CVD = Cardiovascular Disease, NHANES = National Health and Nutrition Examination Survey, FLD = Fatty Liver Disease, Ref = reference, Q = quartile, N = number, TyG = triglyceride-glucose, CMI = Cardiometabolic Index, FLI = Fatty Liver Index, HIS = Hepatic Steatosis Index, LRS = Liver Risk Score, HR = hazard ratio, CI = confidence interval, TC = Total cholesterol.

Table S5. AUC of 10-year time ROC of insulin resistance-fatty liver/fibrosis for Cardiovascular Disease Risk Prediction.

Variables	CKM 0		CKM 3	
	CVD mortality	MACE	CVD mortality	MACE
TyG	0.795	0.769	0.730	0.678
TyG + FLI	0.799	0.772	0.736	0.683
TyG + HSI	0.795	0.769	0.735	0.682
TyG + LRS	0.802	0.771	0.740	0.680
CMI	0.795	0.770	0.730	0.678
CMI + FLI	0.799	0.772	0.736	0.683
CMI + HSI	0.795	0.770	0.734	0.681
CMI + LRS	0.802	0.773	0.741	0.680

Note: Model: Adjusted for sex, age, race. Abbreviations: CVD = Cardiovascular Disease, FLD = Fatty Liver Disease, Ref = reference, Q = quartile, N = number, TyG = triglyceride-glucose, CMI = Cardiometabolic Index, FLI = Fatty Liver Index, HIS = Hepatic Steatosis Index, LRS = LiverRisk Score, HR = hazard ratio, CI = confidence interval.

Table S6. Concordance Index (C-index) and Its 95% Confidence Interval of insulin resistance-fatty liver/fibrosis for Cardiovascular Disease Risk Prediction.

**Variables**	**C index (95%CI)**	**C index (95%CI)**
	**CVD mortality**	**MACE**
TyG	0.749 (0.743–0.755)	0.700 (0.697–0.703)
TyG + FLI	0.757 (0.751–0.763)	0.707 (0.704–0.710)
TyG + HSI	0.754 (0.748–0.760)	0.706 (0.703–0.709)
TyG + LRS	0.757 (0.751–0.763)	0.702 (0.699–0.705)
CMI	0.750 (0.744–0.756)	0.701 (0.698–0.704)
CMI + FLI	0.757 (0.751–0.763)	0.708 (0.705–0.711)
CMI + HSI	0.754 (0.748–0.760)	0.706 (0.703–0.709)
CMI + LRS	0.759 (0.753–0.765)	0.703 (0.700–0.706)

Note: Model: Adjusted for sex, age, race. Abbreviations: CVD = Cardiovascular Disease, FLD = Fatty Liver Disease, Ref = reference, Q = quartile, N = number, TyG = triglyceride-glucose, CMI = Cardiometabolic Index, FLI = Fatty Liver Index, HIS = Hepatic Steatosis Index, LRS = LiverRisk Score, HR = hazard ratio, CI = confidence interval.

Table S7. Joint associations of insulin resistance and fatty liver/fibrosis indices with cardiovascular events in individuals with Cardiovascular-Kidney-Metabolic syndrome stages 0–3 in the UK Biobank.

Variable	N (events)	HR (95% CI)	P value	P for interaction
**CVD mortality**				
**TyG+FLI**				0.619
low-index&low-TyG	97,546 (1204)	1	Ref	
high-index&high-TyG	97,545 (2897)	1.33 (1.23,1.42)	<0.001	
low-index&high-TyG	32,304 (542)	1.08 (0.97,1.19)	0.156	
high-index&low-TyG	32,304 (903)	1.35 (1.23,1.47)	<0.001	
**TyG+HSI**				0.009
low-index&low-TyG	84,792 (1224)	1	Ref	
high-index&high-TyG	84,791 (2362)	1.23 (1.15,1.32)	<0.001	
low-index&high-TyG	45,058 (1077)	1.12 (1.03,1.21)	0.008	
high-index&low-TyG	45,058 (883)	1.16 (1.06,1.26)	0.001	
**TyG+LRS**				0.003
low-index&low-TyG	75,536 (733)	1	Ref	
high-index&high-TyG	75,535 (2546)	1.34 (1.22,1.47)	<0.001	
low-index&high-TyG	54,314 (893)	1.20 (1.09,1.33)	<0.001	
high-index&low-TyG	54,314 (1374)	1.24 (1.12,1.36)	<0.001	
**CMI+FLI**				0.147
low-index&low-CMI	104,963 (1287)	1	Ref	
high-index&high-CMI	104,962 (3117)	1.37 (1.28,1.47)	<0.001	
low-index&high-CMI	24,887 (459)	1.20 (1.08,1.34)	0.001	
high-index&low-CMI	24,887 (683)	1.34 (1.22,1.47)	<0.001	
**CMI+HSI**				0.015
low-index&low-CMI	90,492 (1280)	1	Ref	
high-index&high-CMI	90,491 (2555)	1.28 (1.19,1.37)	<0.001	
low-index&high-CMI	39,358 (1021)	1.18 (1.08,1.28)	<0.001	
high-index&low-CMI	39,358 (690)	1.09 (1.00,1.20)	0.060	
**CMI+LRS**				<0.001
low-index&low-CMI	77,520 (704)	1	Ref	
high-index&high-CMI	77,519 (2654)	1.49 (1.36,1.64)	<0.001	
low-index&high-CMI	52,330 (922)	1.40 (1.26,1.54)	<0.001	
high-index&low-CMI	52,330 (1266)	1.32 (1.20,1.46)	<0.001	
**MACE**				
**TyG+FLI**				0.100
low-index&low-TyG	97,546 (5481)	1	Ref	
high-index&high-TyG	97,545 (12,339)	1.33 (1.28,1.38)	<0.001	
low-index&high-TyG	32,304 (2417)	1.06 (1.01,1.11)	0.028	
high-index&low-TyG	32,304 (3618)	1.32 (1.27,1.38)	<0.001	

Continued on next page

Variable	N (events)	HR (95% CI)	P value	P for interaction
**CVD mortality**				
**TyG+HSI**				0.864
low-index&low-TyG	84,792 (5271)	1	Ref	
high-index&high-TyG	84,791 (10,326)	1.29 (1.25,1.33)	<0.001	
low-index&high-TyG	45,058 (4430)	1.09 (1.05,1.14)	<0.001	
high-index&low-TyG	45,058 (3828)	1.19 (1.15,1.25)	<0.001	
**TyG+LRS**				<0.001
low-index&low-TyG	75,536 (3729)	1	Ref	
high-index&high-TyG	75,535 (10,009)	1.24 (1.19,1.29)	<0.001	
low-index&high-TyG	54,314 (4747)	1.24 (1.18,1.29)	<0.001	
high-index&low-TyG	54,314 (5370)	1.15 (1.10,1.21)	<0.001	
**CMI+FLI**				<0.001
low-index&low-CMI	104,963 (5834)	1	Ref	
high-index&high-CMI	104,962 (13,275)	1.38 (1.33,1.42)	<0.001	
low-index&high-CMI	24,887 (2064)	1.19 (1.13,1.25)	<0.001	
high-index&low-CMI	24,887 (2682)	1.31 (1.25,1.37)	<0.001	
**CMI+HSI**				0.013
low-index&low-CMI	90,492 (5479)	1	Ref	
high-index&high-CMI	90,491 (11,117)	1.35 (1.30,1.39)	<0.001	
low-index&high-CMI	39,358 (4222)	1.17 (1.13,1.22)	<0.001	
high-index&low-CMI	39,358 (3037)	1.15 (1.10,1.21)	<0.001	
**CMI+LRS**				<0.001
low-index&low-CMI	77,520 (3657)	1	Ref	
high-index&high-CMI	77,519 (10,520)	1.36 (1.30,1.42)	<0.001	
low-index&high-CMI	52,330 (4819)	1.38 (1.32,1.44)	<0.001	
high-index&low-CMI	52,330 (4859)	1.19 (1.14,1.25)	<0.001	

Note: Model: adjusted for sex, age, race, education level, Townsend index, smoking status, daily alcohol consumption, hypertension, dyslipidemia, antihypertensive, antidiabetic, lipid-lowering medications. P-values less than 0.05 ($p < 0.05$) were considered significant. Abbreviations: CVD = Cardiovascular Disease, MACE = major adverse cardiovascular events, Ref = reference, Q = quartile, N = number, TyG = triglyceride-glucose, CMI = Cardiometabolic Index, FLI = Fatty Liver Index, HIS = Hepatic Steatosis Index, LRS = Liver Risk Score, HR = hazard ratio, CI = confidence interval.

Table S8. Associations of insulin resistance-fatty liver/fibrosis indices with CVD mortality and MACE in individuals with Cardiovascular-Kidney-Metabolic syndrome stages 0, 1, 2, 3 in the UK Biobank in model 2.

Variable	Stage 0		Stage 1		Stage 2		Stage 3	
	MACE							
	HR (95% CI)	P value	HR (95% CI)	P value	HR (95% CI)	P value	HR (95% CI)	P value
**TyG_FLI**								
T1	1	Ref	1	Ref	1	Ref	1	Ref
T2	1.26 (1.10,1.44)	0.001	1.13 (1.02,1.25)	0.016	1.17 (1.11,1.23)	<0.001	1.13 (1.06,1.20)	<0.001
T3	1.33 (1.16,1.52)	<0.001	1.32 (1.20,1.46)	<0.001	1.36 (1.30,1.43)	<0.001	1.22 (1.14,1.30)	<0.001
**TyG_HSI**								
T1	1	Ref	1	Ref	1	Ref	1	Ref
T2	0.98 (0.88,1.09)	0.677	1.02 (0.94,1.11)	0.628	1.10 (1.05,1.15)	<0.001	1.03 (0.96,1.09)	0.446
T3	1.02 (0.91,1.13)	0.78	1.16 (1.06,1.26)	0.001	1.25 (1.19,1.31)	<0.001	1.12 (1.05,1.20)	0.001
**TyG_LRS**								
T1	1	Ref	1	Ref	1	Ref	1	Ref
T2	1.24 (1.09,1.41)	0.001	1.02 (0.92,1.12)	0.753	1.04 (0.99,1.09)	0.146	0.91 (0.85,0.98)	0.008
T3	1.28 (1.12,1.47)	<0.001	1.05 (0.95,1.17)	0.360	1.14 (1.08,1.21)	<0.001	0.92 (0.86,0.99)	0.034
**CMI_FLI**								
T1	1	Ref	1	Ref	1	Ref	1	Ref
T2	1.27 (1.11,1.44)	<0.001	1.08 (0.98,1.20)	0.107	1.20 (1.14,1.26)	<0.001	1.13 (1.06,1.20)	<0.001
T3	1.34 (1.17,1.53)	<0.001	1.29 (1.18,1.43)	<0.001	1.37 (1.30,1.43)	<0.001	1.19 (1.12,1.27)	<0.001
**CMI_HSI**								
T1	1	Ref	1	Ref	1	Ref	1	Ref
T2	1.14 (1.02,1.28)	0.024	0.97 (0.88,1.06)	0.487	1.23 (1.17,1.29)	<0.001	1.11 (1.04,1.19)	0.001
T3	1.18 (1.05,1.32)	0.005	1.16 (1.07,1.27)	0.001	1.34 (1.28,1.41)	<0.001	1.18 (1.10,1.25)	<0.001

Continued on next page

Variable	Stage 0		Stage 1		Stage 2		Stage 3	
	**MACE**							
	**HR (95% CI)**	**P value**	**HR (95% CI)**	**P value**	**HR (95% CI)**	**P value**	**HR (95% CI)**	**P value**
**CMI_LRS**								
T1	1	Ref	1	Ref	1	Ref	1	Ref
T2	1.27 (1.12,1.44)	<0.001	1.04 (0.94,1.14)	0.465	1.21 (1.15,1.27)	<0.001	1.06 (1.00,1.13)	0.061
T3	1.40 (1.24,1.59)	<0.001	1.17 (1.07,1.29)	0.001	1.34 (1.27,1.40)	<0.001	1.12 (1.06,1.20)	<0.001
	**CVD Mortality**							
**TyG_FLI**								
T1	1	Ref	1	Ref	1	Ref	1	Ref
T2	1.22 (0.89,1.66)	0.223	1.13 (0.89,1.42)	0.314	1.04 (0.93,1.15)	0.507	1.00 (0.87,1.14)	0.982
T3	1.35 (0.98,1.85)	0.063	1.33 (1.06,1.67)	0.014	1.40 (1.27,1.55)	<0.001	1.28 (1.12,1.45)	<0.001
**TyG_HSI**								
T1	1	Ref	1	Ref	1	Ref	1	Ref
T2	0.91 (0.72,1.15)	0.450	1.09 (0.90,1.33)	0.356	0.97 (0.89,1.07)	0.544	0.83 (0.72,0.95)	0.006
T3	0.76 (0.60,0.98)	0.032	1.10 (0.90,1.34)	0.348	1.13 (1.03,1.24)	0.010	1.11 (0.97,1.27)	0.137
**TyG_LRS**								
T1	1	Ref	1	Ref	1	Ref	1	Ref
T2	1.01 (0.74,1.38)	0.948	0.93 (0.73,1.17)	0.521	1.04 (0.93,1.16)	0.503	0.81 (0.69,0.95)	0.008
T3	1.25 (0.92,1.70)	0.149	0.92 (0.73,1.17)	0.515	1.22 (1.09,1.36)	<0.001	0.96 (0.82,1.11)	0.574
**CMI_FLI**								
T1	1	Ref	1	Ref	1	Ref	1	Ref
T2	1.22 (0.90,1.65)	0.204	1.13 (0.90,1.41)	0.296	1.10 (0.99,1.21)	0.074	1.07 (0.94,1.22)	0.292
T3	1.33 (0.98,1.81)	0.064	1.31 (1.05,1.64)	0.016	1.33 (1.21,1.46)	<0.001	1.18 (1.03,1.34)	0.014

Continued on next page

Variable	Stage 0		Stage 1		Stage 2		Stage 3	
	MACE							
	HR (95% CI)	P value	HR (95% CI)	P value	HR (95% CI)	P value	HR (95% CI)	P value
**CMI_HSI**								
T1	1	Ref	1	Ref	1	Ref	1	Ref
T2	0.92 (0.71,1.18)	0.491	0.79 (0.65,0.98)	0.028	1.07 (0.97,1.18)	0.151	0.99 (0.88,1.13)	0.928
T3	0.89 (0.70,1.14)	0.356	1.03 (0.85,1.25)	0.758	1.22 (1.11,1.34)	<0.001	1.09 (0.96,1.24)	0.185
**CMI_LRS**								
T1	1	Ref	1	Ref	1	Ref	1	Ref
T2	1.19 (0.90,1.59)	0.221	0.96 (0.77,1.20)	0.717	1.09 (0.99,1.21)	0.087	1.04 (0.91,1.18)	0.570
T3	1.20 (0.90,1.59)	0.222	1.15 (0.93,1.42)	0.210	1.24 (1.13,1.37)	<0.001	1.13 (1.00,1.29)	0.055

Note: Model 2: adjusted for sex, age, race, education level, Townsend index, smoking status, daily alcohol consumption, hypertension, dyslipidemia, antihypertensive, antidiabetic, lipid-lowering medications. P-values less than 0.05 ($p < 0.05$) were considered significant. Abbreviations: CVD = Cardiovascular Disease, FLD = Fatty Liver Disease, Ref = reference, Q = quartile, N = number, TyG = triglyceride-glucose, CMI = Cardiometabolic Index, FLI = Fatty Liver Index, HIS = Hepatic Steatosis Index, LRS = LiverRisk Score, HR = hazard ratio, CI = confidence interval.

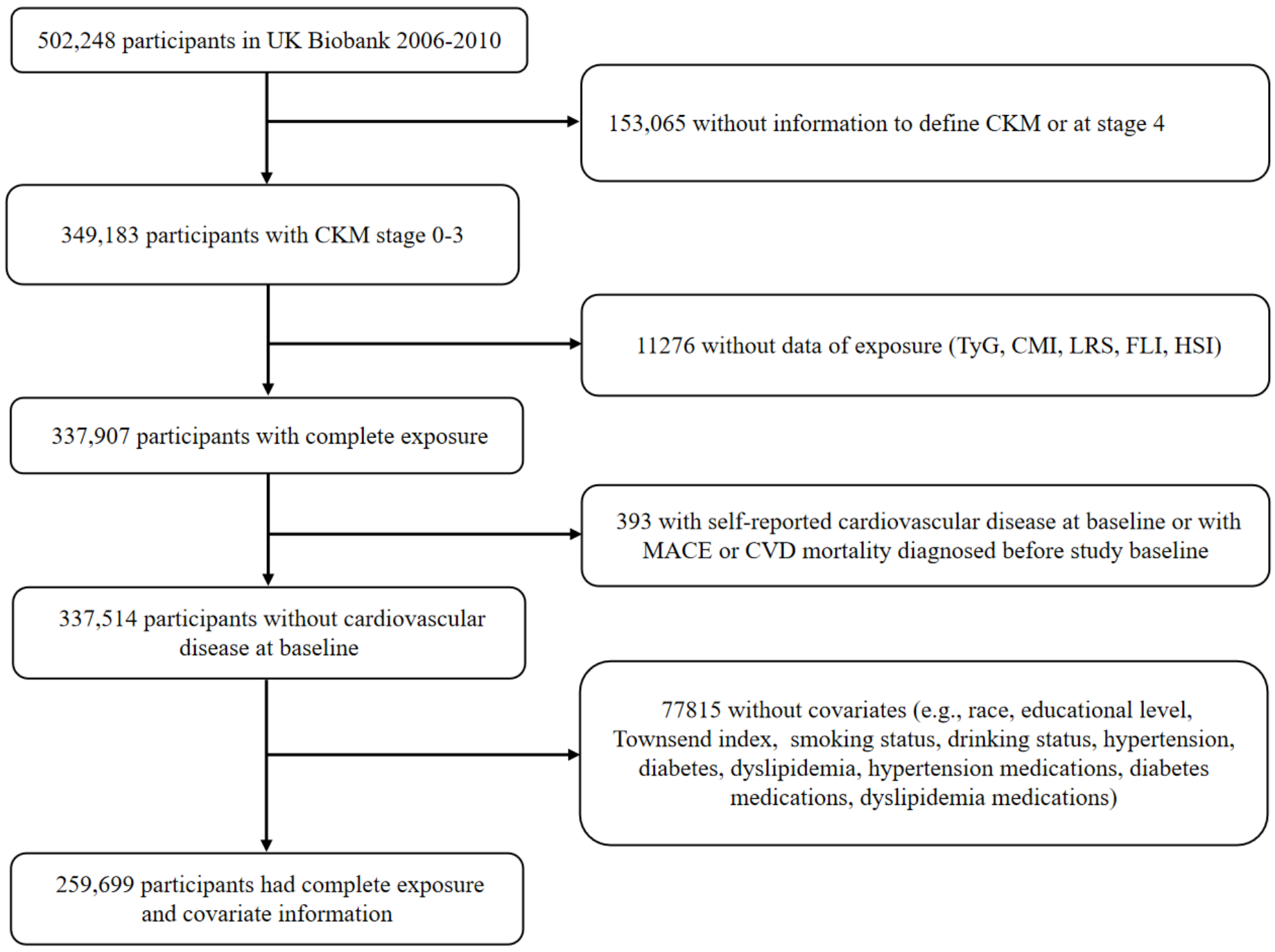

Figure S1. Flow chart of UK Biobank.

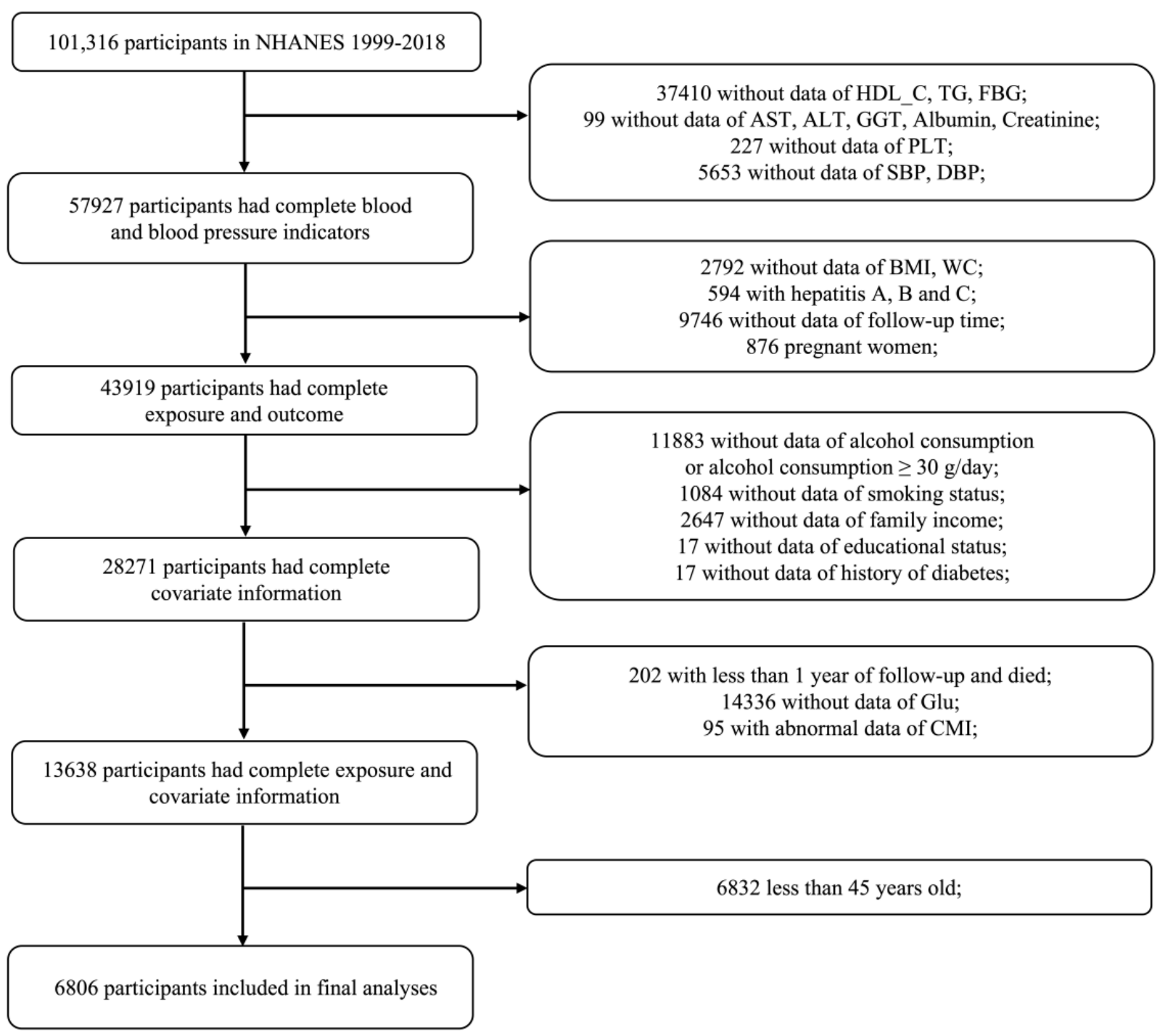

Figure S2. Flow chart of NHANES. Abbreviations: NHANES=National Health and Nutrition Examination Survey.

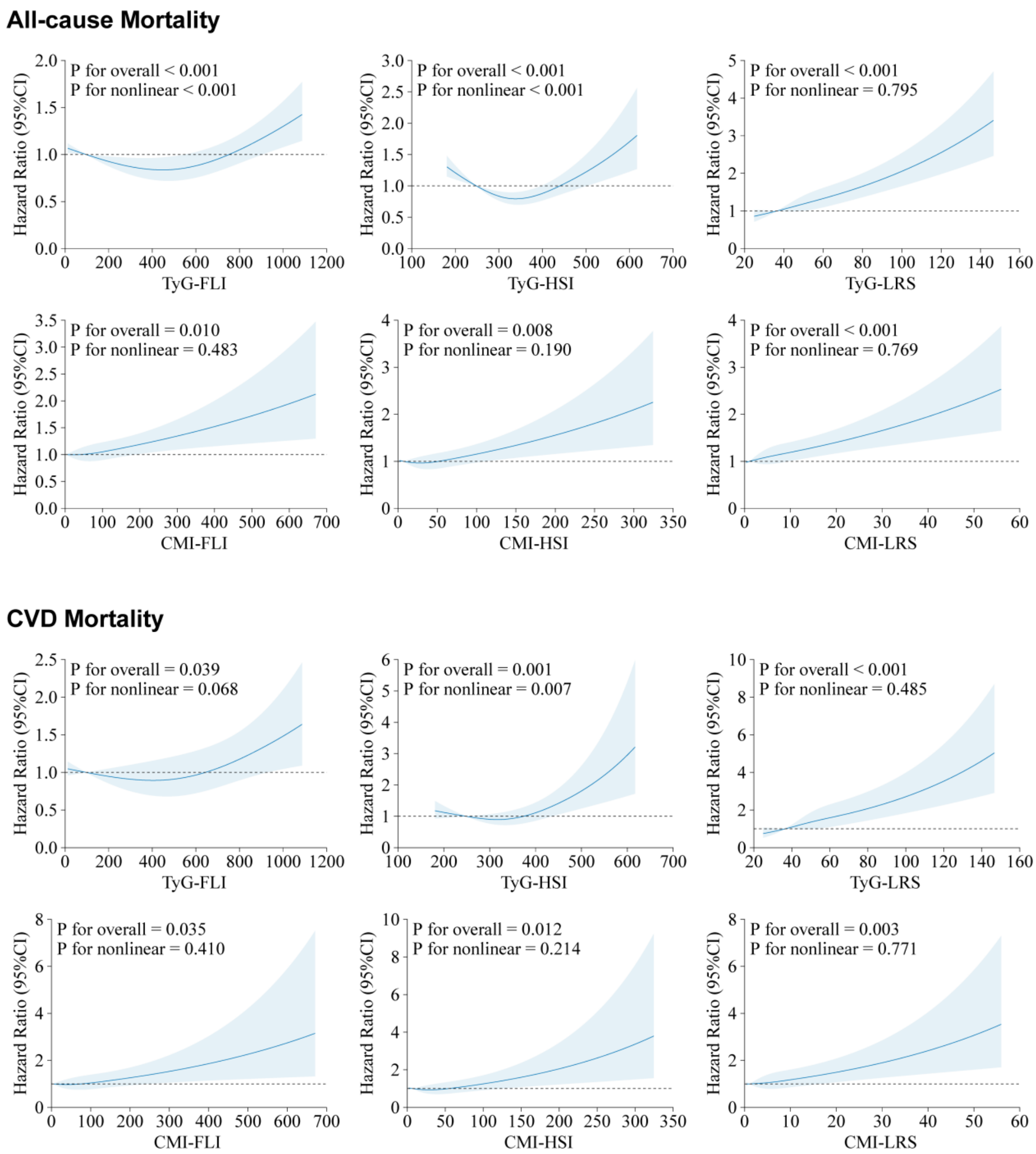

Figure S3. Restricted cubic spline curve illustrating the associations of insulin resistance-fatty liver/fibrosis indices with all-cause and CVD mortality in the NHANES. Note: The Model was adjusted for sex, age, race, education level, income level, smoking status, daily alcohol consumption, hypertension, TC. P-values less than 0.05 ($p < 0.05$) were considered significant. Abbreviations: NHANES = National Health and Nutrition Examination Survey, CVD = Cardiovascular Disease, Ref = reference, Q = quartile, N = number, TyG = triglyceride-glucose, CMI = Cardiometabolic Index, FLI = Fatty Liver Index, HIS = Hepatic Steatosis Index, LRS = Liver Risk Score, HR = hazard ratio, CI = confidence interval, TC = Total cholesterol.

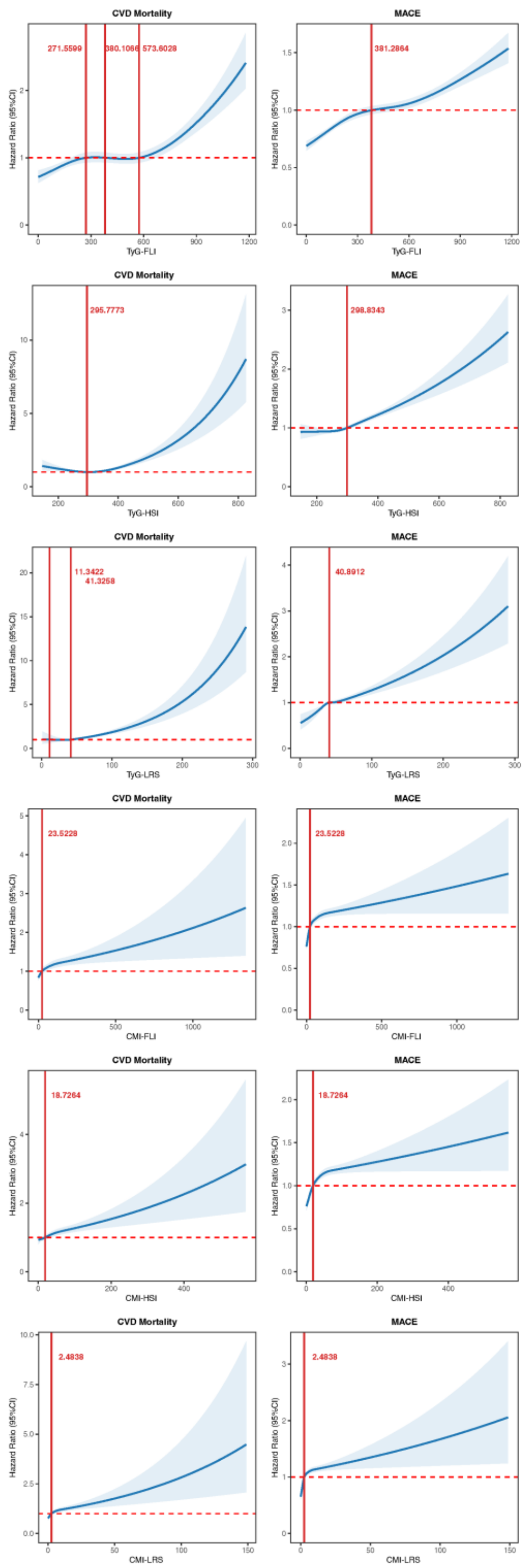

Figure S4. Restricted cubic spline curve illustrating the associations of insulin resistance-fatty liver/fibrosis indices with MACE and CVD mortality in the UK Biobank. Note: The model was adjusted for sex, age, race, education level, income level, smoking status, daily alcohol consumption, hypertension, TC. Abbreviations: CVD = Cardiovascular Disease, MACE = major adverse cardiovascular events, TyG = triglyceride-glucose, CMI = Cardiometabolic Index, FLI = Fatty Liver Index, HIS = Hepatic Steatosis Index, LRS = LiverRisk Score, HR = hazard ratio, TC = Total cholesterol.

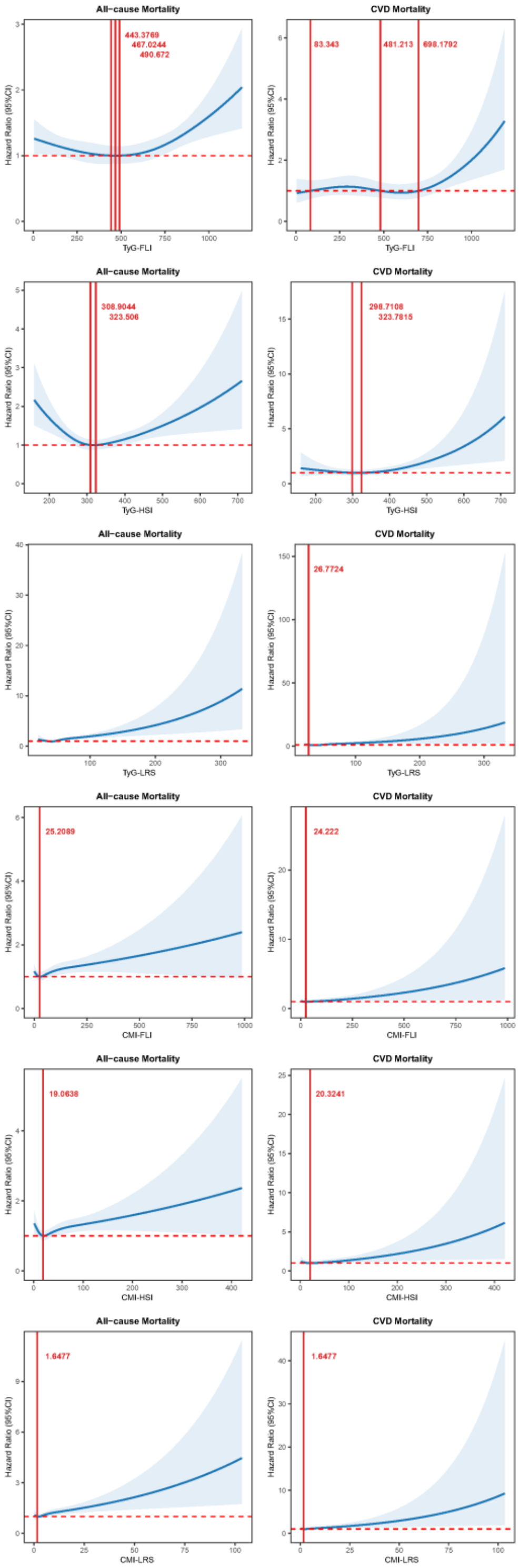

Figure S5. Restricted cubic spline curve illustrating the associations of insulin resistance-fatty liver/fibrosis indices with MACE and CVD mortality in the NHANES. Note: The model was adjusted for sex, age, race, education level, income level, smoking status, daily alcohol consumption, hypertension, TC. Abbreviations: CVD = Cardiovascular Disease, MACE = major adverse cardiovascular events, TyG = triglyceride-glucose, CMI = Cardiometabolic Index, FLI = Fatty Liver Index, HIS = Hepatic Steatosis Index, LRS = LiverRisk Score, HR = hazard ratio, TC = Total cholesterol.

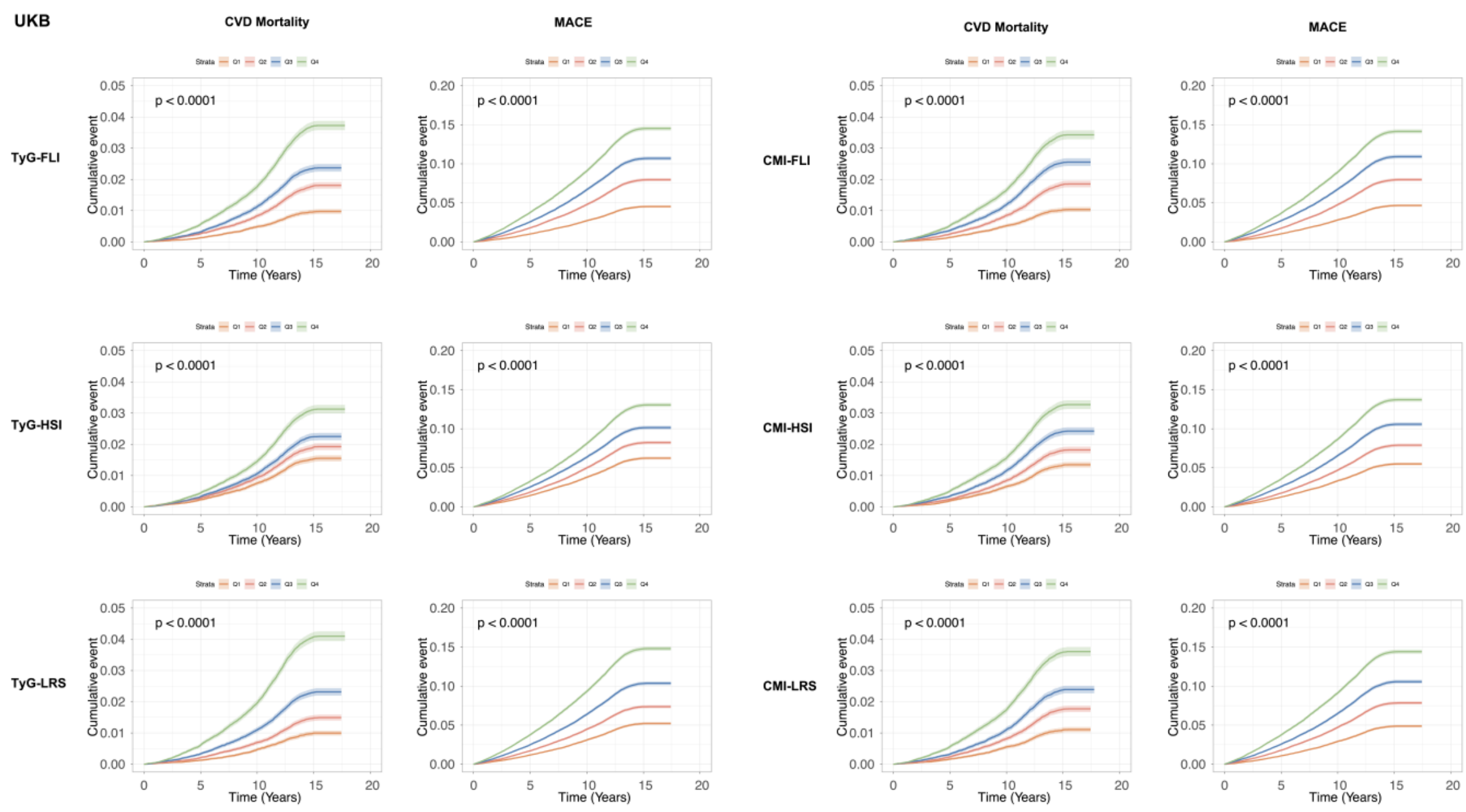

Figure S6. Cumulative incidence of CVD mortality and MACE stratified by insulin resistance-fatty liver/fibrosis indices: A Kaplan-Meier analysis in the UK Biobank. Note: P-values less than 0.05 ($p < 0.05$) were considered significant. Abbreviations: CVD = Cardiovascular Disease, MACE = major adverse cardiovascular events, Ref = reference, Q = quartile, N = number, TyG = triglyceride-glucose, CMI = Cardiometabolic Index, FLI = Fatty Liver Index, HIS = Hepatic Steatosis Index, LRS = Liver Risk Score, HR = hazard ratio, CI = confidence interval.

AIMS Press

© 2026 the Author(s), licensee AIMS Press. This is an open access article distributed under the terms of the Creative Commons Attribution License (http://creativecommons.org/licenses/by/4.0)
